# Everolimus, a mammalian target of rapamycin inhibitor, ameliorated streptozotocin-induced learning and memory deficits via neurochemical alterations in male rats

**DOI:** 10.17179/excli2018-1626

**Published:** 2018-10-29

**Authors:** Sahar Fanoudi, Mahmoud Hosseini, Mohaddeseh Sadat Alavi, Mohammad Taher Boroushaki, Azar Hosseini, Hamid R. Sadeghnia

**Affiliations:** 1 Department of Pharmacology, Faculty of Medicine, Mashhad University of Medical Sciences, Mashhad, Iran; 2 Pharmacological Research Center of Medicinal Plants, Mashhad University of Medical Sciences, Mashhad, Iran; 3 Division of Neurocognitive Sciences, Psychiatry and Behavioral Sciences Research Center, Mashhad University of Medical Sciences, Mashhad, Iran

**Keywords:** everolimus, mTOR, Alzheimer's disease (AD), oxidative stress, acetylcholinesterase, streptozotocin

## Abstract

Everolimus (EVR), as a rapamycin analog, is a selective inhibitor of the mammalian target of rapamycin (mTOR) kinase and its associated signaling pathway. mTOR is a serine/threonine protein kinase and its hyperactivity is involved in the pathophysiology of Alzheimer's disease (AD) and associated cognitive deficits. The present study evaluated the impact of EVR, on cognitive functions, hippocampal cell loss, and neurochemical parameters in the intracerebroventricular streptozotocin (icv-STZ) model of AD rats. EVR (1 and 5 mg/kg) was administered for 21 days following the single administration of STZ (3 mg/kg, icv) or for 7 days on days 21-28 post-STZ injection after establishment of cognitive dysfunction. Cognitive deficits (passive avoidance and spatial memory), oxidative stress parameters, acetylcholinesterase (AChE) activity, and percentage of cell loss were evaluated in the hippocampus. Chronic administration (1 and 5 mg/kg for 21 days from the day of surgery and icv-STZ infusion) or acute injection (5 mg/kg for 7 days after establishment of cognitive impairment) of EVR significantly attenuated cognitive dysfunction, neuronal loss, oxidative stress and AChE activity in the hippocampus of STZ-AD rats. In conclusion, our study showed that EVR could prevent or improve deteriorations in behavioral, biochemical and histopathological features of the icv-STZ rat model of AD. Therefore, inhibition of the hyperactivated mTOR may be an important therapeutic target for AD.

## Introduction

The most prevalent type of dementia is Alzheimer's disease (AD), which is considered as a debilitating neurodegenerative illness, characterized by the progressive cognitive impairments and extensive death of neurons mainly of the cholinergic system. The presence of senile plaques derived from amyloid beta (Aβ) protein and neurofibrillary tangles (NFT), particularly in the hippocampus or cerebral cortex, are the hallmarks of AD (Ebrahimi et al., 2017[25]). At the present time, the most commonly available medications for AD treatment are acetylcholinesterase (AChE) inhibitors (tacrine, donepezil, rivastigmine and galantamine), which are aimed to improve cognition without any disease modifying properties (Casey et al., 2010[16]; Stuchbury and Münch, 2005[76]). Therefore, identifying novel therapeutic strategies that prevent or reverse the underlying pathological mechanisms of AD are needed. 

The mammalian target of rapamycin (mTOR) is a serine/threonine protein kinase that has a key role in various cellular functions. mTOR signaling dysfunction has been involved in the pathophysiology of several neurodegenerative, neurological, and neurodevelopmental disorders (Perluigi et al., 2015[57]; Russo et al., 2012[64]; Wong, 2013[88]). mTOR signaling is necessary for memory retention, synaptic plasticity, neuroendocrine regulation, and neuronal recovery in the central nervous system (CNS) (Blagosklonny, 2012[8]; Chong et al., 2010[18]; Pei and Hugon, 2008[56]; Troca-Marín et al., 2012[83]). However, mTOR hyperactivity is closely associated with the presence of two hallmarks of AD (Aβ plaques and NFT) and cognitive deficits in clinical presentation (Cai et al., 2012[14]; Lafay-Chebassier et al., 2005[41]; Pozueta et al., 2013[60]). The intracerebroventricular (icv) injection of the sub-diabetogenic dose of streptozotocin (STZ) (at a dose of 3 mg/kg) provides a related model for sporadic dementia of AD (Ramezani et al., 2016[61]). It has been reported that icv-STZ induced brain insulin resistance, reduced brain glucose metabolism, oxidative stress, cholinergic deficits, gliosis, accumulation of tau and Aβ proteins, and learning and memory impairments in rats (Kamat, 2015[36]; Salkovic-Petrisic et al., 2013[67]). In addition, it has been shown that the activity of the hippocampal mTOR system increased two weeks post icv-STZ injection (An et al., 2003[3]; Wang et al., 2014[86]). Therefore, central administration of STZ in rats caused behavioral, histological, and neurochemical changes like human AD features (Dhull et al., 2012[24]; Ponce-Lopez et al., 2011[59]). 

Everolimus (EVR), as a rapamycin analog, is a selective mTOR inhibitor, mainly mTORC1 and likely mTORC2, which are two main complexes of mTOR (Hasskarl, 2014[30]; Sarbassov et al., 2006[69]). EVR is clinically used as an immunosuppressive drug in organ transplantation and also as a medication in human cancer treatment (Richardson et al., 2015[63]; Yates, 2016[89]). In a human study on recipients of heart transplant, EVR improved psychiatric symptoms and memory (Lang et al., 2009[43]). Furthermore, rapamycin administration improved memory functions in murine models of AD (Caccamo et al., 2010[13]; Majumder et al., 2012[48]; Spilman et al., 2010[75]). The present study was designed to investigate, for the first time, the effects of EVR as a selective mTOR inhibitor on learning and memory deficits, hippocampal oxidative stress, AChE activity, and neuronal loss in STZ-induced AD rats.

## Material and Methods

### Chemicals

5,5´-Dithiobis-2-nitrobenzoic acid (DTNB), 2-thiobarbituric acid (TBA), HCl, trichloroacetic acid (TCA), ethylenediaminetetraacetic acid disodium salt (Na_2_EDTA), tris (hydroxymethyl) aminomethane (Trizma base), phosphate buffered saline (PBS), and dimethyl sulfoxide (DMSO) were purchased from Merck (Darmstadt, Germany). Everolimus (EVR), streptozotocin, and acetylthiocholine iodide were purchased from Sigma (St. Louis, USA).

### Animals

In this study, male Wistar rats weighing 200 ± 20 g (6-8 weeks of age) at the beginning of the experiments were used. The animals were obtained from the Animal House of the Faculty of Medicine, Mashhad University of Medical Sciences, Mashhad, Iran. The animals were kept 4 to 5 per cage under standard temperature (22 ± 2 °C), and lighting (12 h light/dark cycle) conditions for all the trials. The rats had *ad libitum* access to food and water. All animals were treated in accordance with the National Institutes of Health Guidance for the Care and Use of Laboratory Animals and the Animal Ethics Committee of the Mashhad University of Medical Sciences approved the experimental protocols.

### Experimental protocols

**Figure 1:**
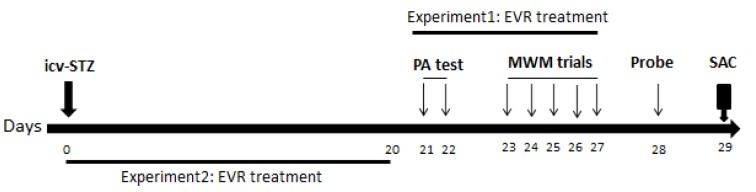
Diagrammatic sketch for the behavioral, biochemical and histopathological experiments. EVR: everolimus; icv-STZ: intracerebroventricular-streptozotocin; PA: passive avoidance; MWM: Morris water maze; SAC: sacrificed for biochemical or histopathological experiments. Day 0 refers to the day of surgery (icv-STZ infusion).

In the present study, two experimental protocols were used (Figure 1(Fig. 1)). Experiment I was done to determine the therapeutic effect of EVR on STZ-induced AD (groups 1-4). To do so, EVR was intraperitoneally (ip) injected to AD rats on days 21-28 post-STZ injection, one hour before the behavioral study. As our preliminary experiments, along with other studies, showed that learning and memory impairments were established at day 21 post-STZ administration (Ashrafpour et al., 2015[5]; Mehla et al., 2013[50]; Salkovic-Petrisic et al., 2013[67]). Experiment II was carried out to evaluate the effect of the mTORC1 inhibitor on the progress of Alzheimer's disease (groups 1, 2, 5-7). Therefore, EVR was administered from day 0 [day of icv-STZ injection] to day 20 (Veerendra Kumar and Gupta, 2003[85]). 

After acclimatization, the rats were randomly divided into 7 groups (n = 9-12): 

Group 1 - sham (icv administration of normal saline + ip injection of vehicle solution)

Group 2 - STZ (STZ 3 mg/kg was administered once, icv)

Group 3 - STZ + EVR 1 mg/kg (for 7 days)

Group 4 - STZ + EVR 5 mg/kg (for 7 days)

Group 5 - STZ + EVR 1 mg/kg (for 21 days)

Group 6 - STZ + EVR 5 mg/kg (for 21 days) 

Group 7 - EVR 5 mg/kg (for 21 days).

Passive avoidance paradigm on days 21-22 and Morris water maze test on days 23-28 were done. Twenty-four hours after probe test, the animals were euthanized and their brains were quickly removed. Then the hippocampus was separated and dissected on a glass plate located on ice and stored at -80 °C for biochemical assays. The samples were thawed and homogenized (10 % w/v) in ice-cold 0.1 M phosphate buffer (pH 7.4) to determine of oxidative stress parameters and acetylcholinesterase enzyme (AChE) activity. Hematoxylin and eosin (H & E) staining were done for histopathological examinations.

### Surgical procedure: Intracerebroventricular (icv) administration of streptozotocin

For surgical procedure, the rats were anaesthetized using a combination of ketamine (100 mg/kg, i.p.) and xylazine (20 mg/kg, i.p.). Then, the head of the animal was restrained onto a stereotaxic frame, and a midline sagittal incision was made in the scalp and the skull was exposed. Coordinates for the lateral ventricles (LV) were -0.8 mm posterior and 1.5 mm lateral from the bregma and -4.0 mm below (Paxinos and Watson, 1986[55]). Through these skull holes, a 28-gauge stainless steel needle was lowered into each LV, and STZ was injected slowly and bilaterally at a dose of 3 mg/kg (5 µL/side). To prepare the STZ solution, it was freshly dissolved in sterile 0.9 % saline before the injection. The animals in the STZ and STZ + EVR groups received an icv injection of STZ and the sham group received the same volume of sterile saline, icv. After the surgery, the animals were kept separately in individual cages. The stereotaxic coordinates for icv administration was confirmed using an injection of 5 µl methylene blue and anatomical observation 24 h after the injection.

### Behavioral study

Twenty-one days after icv-STZ injection, behavioral tests were started. The experiments were performed at standard optimal conditions between 9:00 am and 4:00 pm in the laboratory (Bassani et al., 2017[7]).

### Passive avoidance test 

The animals underwent a passive avoidance learning test to evaluate their memory retention deficit (Reeta et al., 2009[62]). In this test, the rat was placed in the apparatus, consisting of a light and a dark compartment separated by a guillotine door. On the acquisition trial, the rat was accustomed to the light chamber for 60 s. After that, the guillotine was opened and the initial latency to enter the dark compartment was recorded. Rats that exhibited an initial latency time of more than 60 s were excluded from further experiments. When the rat entered the dark chamber, the guillotine door was closed and an electric foot shock (1 mA, 2 s duration) was delivered through the grid floor. Then, the rat was removed from the dark chamber and returned to the home cage. Three and 24 hour later, retention latency time was measured in the same way, without the foot shock, and the latency time to enter the dark chamber was recorded up to a maximum of 300 s.

### Morris water maze (MWM) test

After the passive avoidance test, the rats were submitted to training in the Morris water maze (MWM) apparatus to test their spatial learning and memory (Morris, 1984[52]). It consisted of a black circular tank (136 cm diameter, 60 cm height) filled with water (depth 30 cm; 24 ± 1 °C) and a circular platform (10 cm diameter) was hidden 2 cm below the surface of the water in the center of the southwest quadrant of the pool. Movement of the animals was recorded by a camera above the pool that was connected to a computer. The pool was divided into four equal quadrants, as four different starting positions, labeled North (N), South (S), East (E), and West (W). All four starting points were utilized once in a random sequence. Before the training, each rat was habituated to MWM for 30 s without the platform. For each animal four trials were carried out daily, for 5 consecutive days. Each trial was started when the rat was released into the tank, facing the wall of tank at one of the four random starting positions. The rat was allowed a maximal time of 60 s for swimming to find the hidden platform and to remain on the platform for 15 s. If the rat failed to find the hidden platform within 60 s, it was gently placed on the platform and remained there for 15 s. After each trial, the rat was dried and at the end of the session it was returned to its cage. The time to reach the platform (latency time) and the length of the swimming path (traveled distance) were recorded in training trials by a video tracking system. At 24 h after the acquisition phase, the platform was removed and probe test was conducted, and the rats were given 60 s to swim to explore the platform. The time spent in the target quadrant and the original position of the platform served as the degree of memory consolidation.

### Biochemical measurements 

#### Tissue preparation

Twenty-four hours after the probe test, the animals were euthanized under deep anesthesia, the brains were quickly taken out, and the hippocampus was separated and dissected on a glass plate located on ice and stored at -80 °C for biochemical assays. Then samples were thawed and homogenized (10 % w/v) in ice-cold 0.1 M phosphate buffer (pH 7.4) to determine oxidative stress parameters, as well as acetylcholinesterase enzyme (AChE) activity.

### Oxidative stress markers

### Estimation of lipid peroxidation

The level of malondialdehyde (MDA) as a lipid peroxidation marker was estimated as described previously (Vafaee et al., 2015[84]; Zabihi et al., 2014[91]). The reaction of MDA with TBA leads to the production of a red complex with a peak absorbance at 535 nm. Briefly, 2 mL of TBA/trichloroacetic acid (TCA)/hydrochloric acid (HCL) reagent was added to 1 mL of the hippocampus homogenate. Then, the solution was placed in a boiling water bath for 40 min. After cooling and centrifugation (1000 g for 10 minutes), the absorbance was measured at 535 nm. The MDA concentration (C) was calculated based on this equation:

C (M) = Absorbance / 1.56 × 10^5^.

#### Estimation of total thiol group

Total thiol groups were estimated using DTNB (2,2'-dinitro-5,5'-dithiodibenzoic acid), a thiol specific reagent. DTNB reacts with the SH groups and generates a yellow mixture with a peak absorbance at 412 nm. Briefly, 50 μl of the homogenate sample was mixed with 1 ml of Tris-EDTA buffer (pH = 8.6) and the absorbance was measured at 412 nm against the Tris-EDTA buffer alone (A1). Then, 20 μl of the DTNB reagents (10 mM in methanol) was added to the mixture and after 15 min (while being kept in laboratory temperature), the sample absorbance was read again (A2). The absorbance of the DTNB reagent was also measured as a blank (B). Total thiol concentration (mM) was calculated according to the following equation (Hosseinzadeh and Sadeghnia, 2005[31]):

Total thiol concentration (mM) = (A-A1-B) × 1.07/0.05 × 13.6.

#### Cholinesterase activity measurement

The AChE activity was measured in rat hippocampus using the method of Ellman et al. (1961[26]). Briefly, 0.1 mL of 10 mM DTNB was added to 0.04 mL of homogenate sample and 2.55 mL of PBS. This solution was stored for 5 min at 37 °C and the absorbance was measured at 412 nm. Then, 0.02 mL of 75 mM acetylthiocholine iodide was added, and further incubated at 37 °C for 5 min. Then, the alteration in the absorbance was read at 412 nm. AChE activity was calculated and reported as μmol/g tissue/min (Ellman et al., 1961[26]; Isomae et al., 2002[33]; Zhong et al., 2009[93]).

#### Histopathological examinations

The animals were deeply anaesthetized and transcardially perfused with 100 mL of heparinized phosphate buffered saline (PBS), followed by 100 mL of 4 % paraformaldehyde in phosphate buffer (pH 7.4). Brains were carefully removed and postfixed in the same fixative for 24 h, dehydrated, and embedded in paraffin using an automated tissue processor. A total number of six coronal brain sections (5 μm) per rat were used. The sections were then stained with H&E and examined under a Leica DMRB microscope (Leica, India). Photographs were then taken using a Canon PowerShot S70 digital camera (Canon, Japan) at 400x magnification, and cell counts in both the ipsilateral and contralateral sides of the hippocampal areas were scored in a blinded manner (Sadeghnia et al., 2017[66]).

### Statistical analysis

GraphPad Prism (version 6.01, La Jolla, CA) software was used for statistical evaluation. All the data were expressed as mean ± SEM. Statistical analysis of the results during the five days of the MWM and passive avoidance tests were carried out using repeated measures analysis of variance (ANOVA) with Tukey's post hoc testing. Moreover, statistical analysis of the probe trial and swimming speed in the MWM, MDA, and total thiol contents and AChE activity were performed using one-way ANOVA followed by Tukey's post hoc testing. A statistical p value < 0.05 was considered significant.

## Results

### EVR improved STZ-induced memory deficits in the passive avoidance test

**Figure 2:**
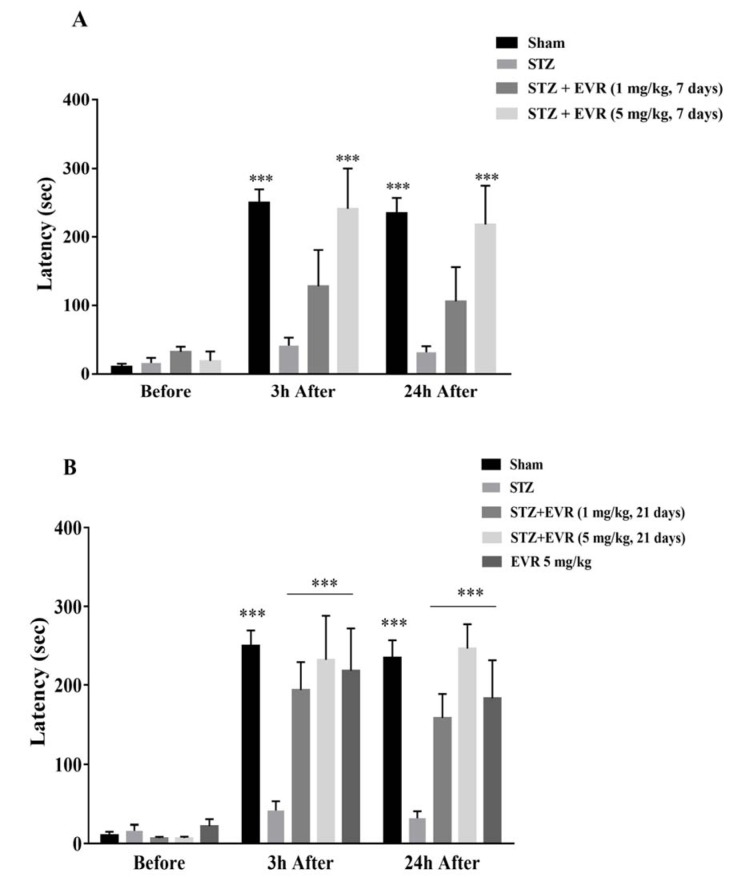
Effects of 7 days (A, on days 21-28 post-STZ infusion, after establishment of cognitive dysfunctions) or 21 days (B, from day 0 which refers to the day of surgery and icv-STZ infusion) administration of EVR on passive avoidance memory of STZ-induced AD rats. The latency to enter the dark chamber of the shuttle box apparatus was acquised before, 3 h and 24 h after delivering electrical foot shock (1 mA, 2 s duration). Values are means ± SEM. ***p < 0.001 *vs.* STZ group.

As shown in Figure 2A-B(Fig. 2), at baseline, there was no significant difference in the mean initial latency time among all groups. However, the retention latency time was significantly decreased (p < 0.001) in the STZ group compared with the sham animals 3 and 24 h after the shock.

Short-term treatment with EVR (5 mg/kg for 7 days) significantly improved retention latency time, as compared with the STZ group (p < 0.001). There was no significant difference in the latency time of the EVR group (1 mg/kg for 7 days) when compared with the STZ group (Figure 2A(Fig. 2)).

However, retention latency was significantly altered towards the control value after the STZ rats were chronically administered with EVR at the doses of 1 mg/kg (p < 0.001) and 5 mg/kg (p < 0.001) for 21 days (Figure 2B(Fig. 2)).

EVR improved STZ-induced learning and memory deficits in the Morris water maze test.

**Figure 3:**
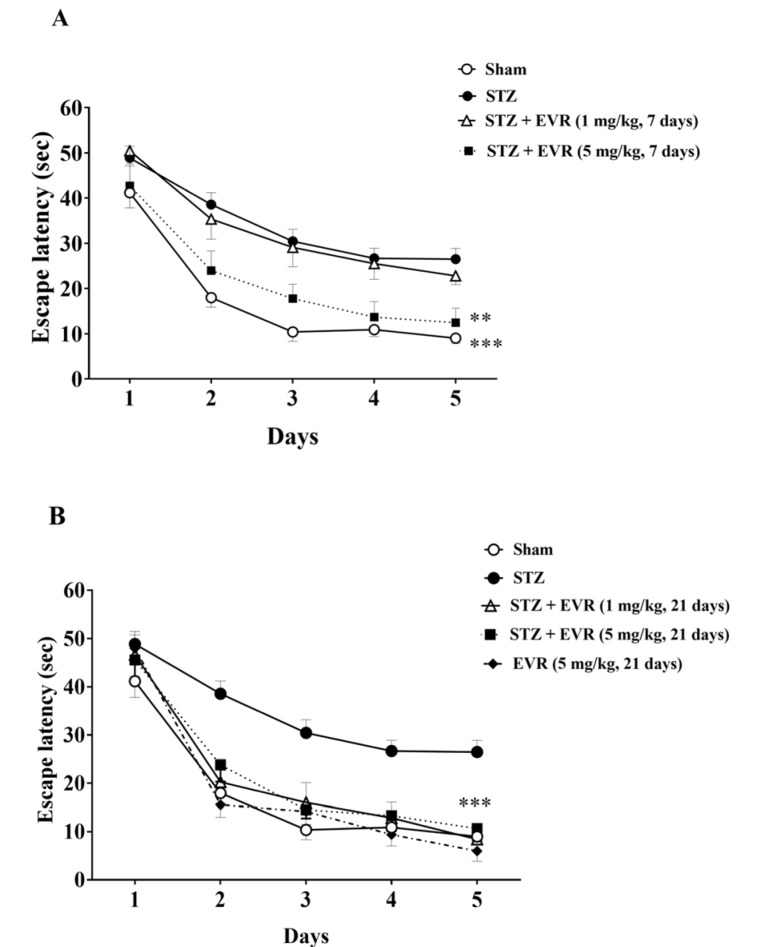
Effects of 7 days (A, on days 21-28 post-STZ infusion, after establishment of cognitive dysfunction) or 21 days (B, from day 0 which refers to the day of surgery and icv-STZ infusion) administration of EVR on escape latency of STZ-induced AD rats in Morris water maze (MWM) task. Values are means ± SEM. **p<0.01 and ***p<0.001 *vs.* STZ group.

**Figure 4:**
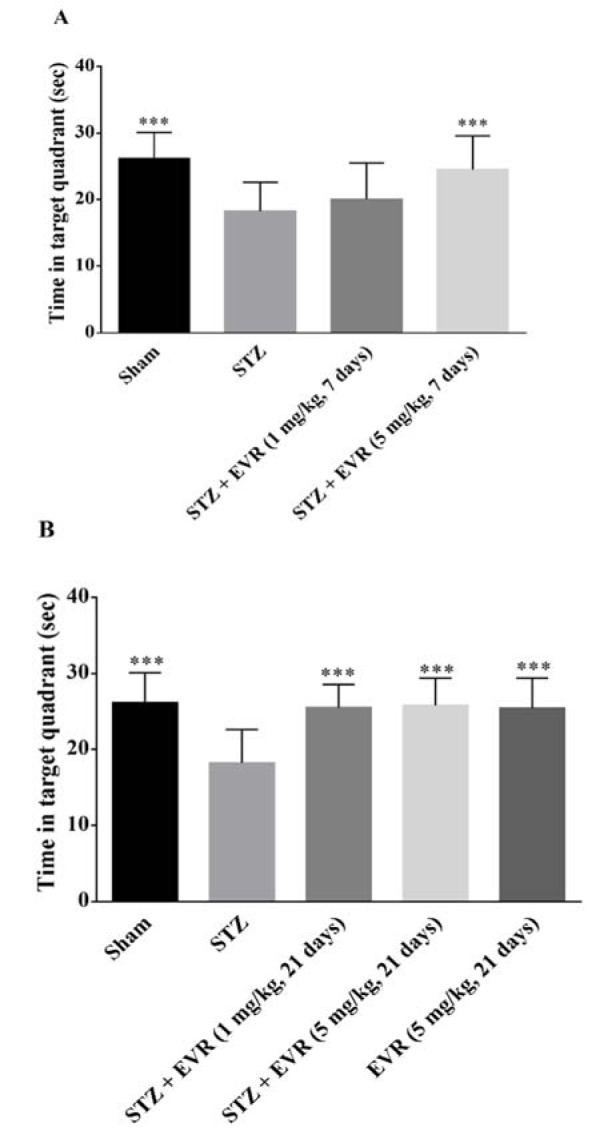
Effects of 7 days (A, on days 21-28 post-STZ infusion, after establishment of cognitive dysfunction) or 21 days (B, from day 0 which refers to the day of surgery and icv-STZ infusion) administration of EVR on time spent in target quadrant of STZ-induced AD rats in Morris water maze (MWM) task. Values are means ± SEM. ***p < 0.001 *vs.* STZ group.

**Figure 5:**
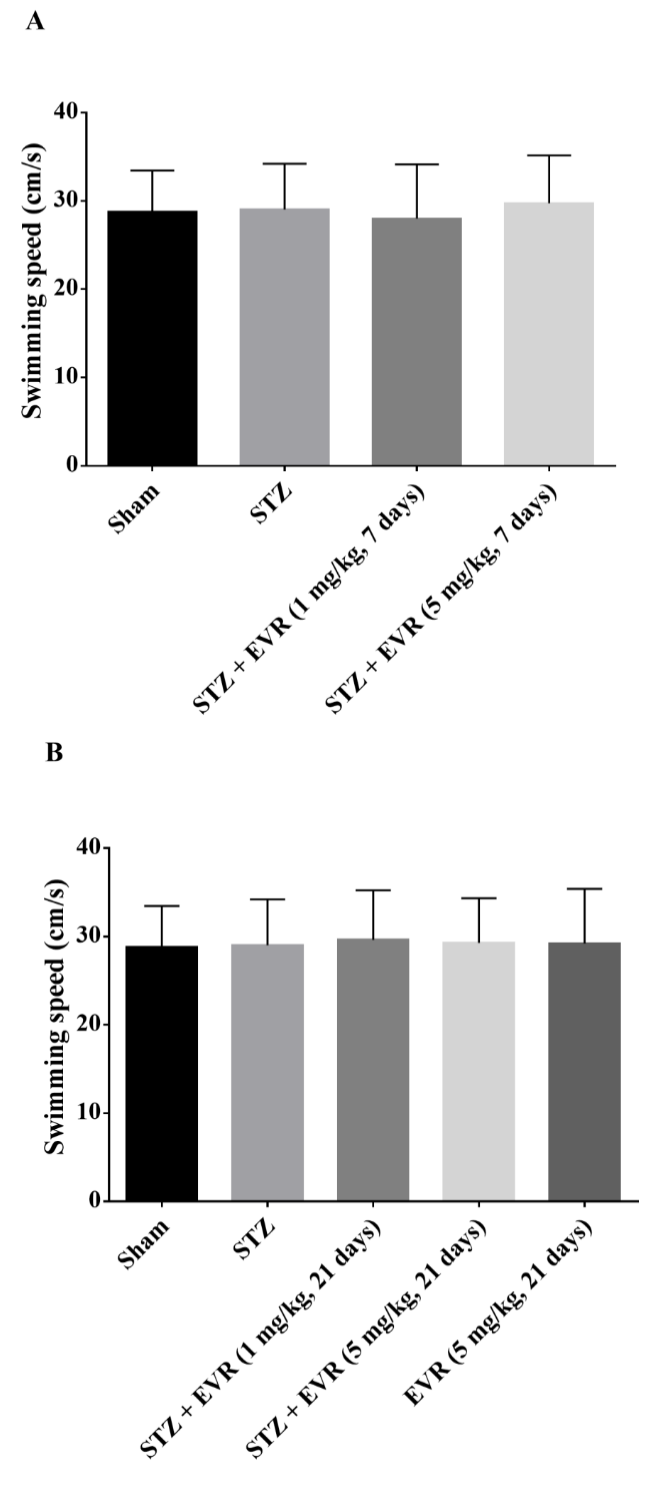
Effects of 7 days (A, on days 21-28 post-STZ infusion, after establishment of cognitive dysfunction) or 21 days (B, from day 0 which refers to the day of surgery and icv-STZ infusion) administration of EVR on swimming speed of STZ-induced AD rats in Morris water maze (MWM) task. Values are means ± SEM. No significant differences were seen between sham-, STZ and EVR-treated rats.

Figure 3(Fig. 3) shows the decrease in latency time to find the hidden platform in all groups during the five-day training trials in the MWM task. According to Figures 3A-B(Fig. 3), STZ rats showed significant higher escape latency than the sham-operated animals from day 2 (p < 0.001) to day 5 (p < 0.001). Results also revealed that short-term treatment with a high dose of EVR (5 mg/kg for 7 days), and not a low dose (1 mg/kg for 7 days), significantly improved learning performance as compared to the STZ group (Figure 3A(Fig. 3), p < 0.05). On the other hand, chronic administration of EVR (1 and 5 mg/kg) for 21 days significantly decreased the mean latency time to find the hidden platform, as compared with the STZ group (p < 0.001) (Figure 3B(Fig. 3)). According to Figs. 4A-B(Fig. 4) on the probe trial, the STZ rats failed to remember the platform location and spent less time in the target quadrant than the sham group (p < 0.001). Figure 4A(Fig. 4) shows that short-term treatment with EVR (5 mg/kg for 7 days) significantly increased the time spent in the target quadrant, as compared to the STZ group (p < 0.001). However, treatment with a low dose of EVR (1 mg/kg for 7 days) failed to improve STZ-induced memory deficits. In the same way, the time spent in the target quadrant was significantly improved following chronic administration of EVR (at both low and high doses), as compared to the STZ group, validating a marked cognitive improvement (Figure 4B(Fig. 4), p < 0.001). Furthermore, there were no significant differences among the swimming speeds of all groups during the experiments, indicating no sensorimotor effect (Figure 5A-B(Fig. 5), p > 0.05).

### Effect of EVR on the hippocampal MDA level

**Figure 6:**
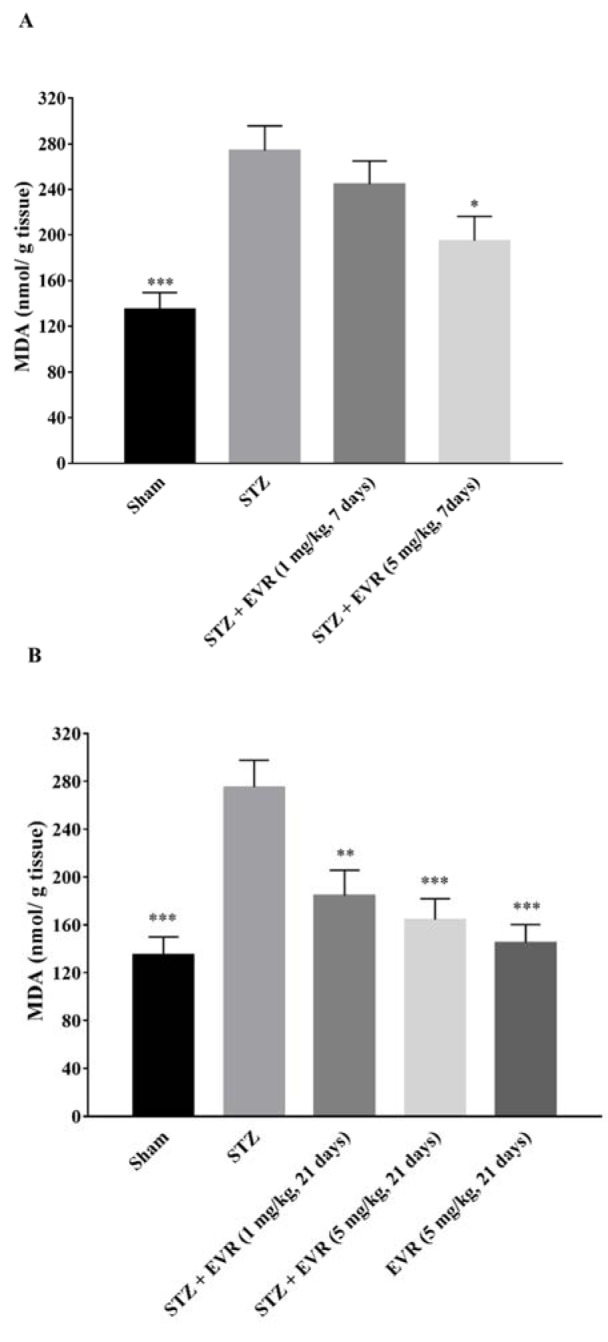
Effects of 7 days (A, on days 21-28 post-STZ infusion, after establishment of cognitive dysfunction) or 21 days (B, from day 0 which refers to the day of surgery and icv-STZ infusion) administration of EVR on malondialdehyde (MDA) level in the hippocampus of STZ-induced AD rats. Values are means ± SEM. *p<0.05, **p<0.01 and ***p < 0.001 *vs.* STZ group.

The degree of free radical damage following the STZ injection was evaluated via lipid peroxidation, which was measured as MDA levels. According to Figure 6(Fig. 6), the hippocampal MDA level was significantly increased in the STZ group (p < 0.001), in comparison to the sham group. 

Treatment with a high dose of EVR (5 mg/kg for 7 days) resulted in a significant reduction in the lipid peroxidation as illustrated by a decrease in the MDA level, in comparison with the STZ group (Figure 6A(Fig. 6), p < 0.05). Chronic administration of EVR also significantly attenuated the increased MDA levels induced by STZ, at both doses of 1 and 5 mg/kg (p < 0.01 and p < 0.001, respectively) in the hippocampus (Figure 6B(Fig. 6)). MDA levels were not changed significantly when EVR (5 mg/kg for 21 days) was given alone, as compared to the sham group (Figure 6B(Fig. 6), p > 0.05) 

### Effect of EVR on hippocampal thiol levels

**Figure 7:**
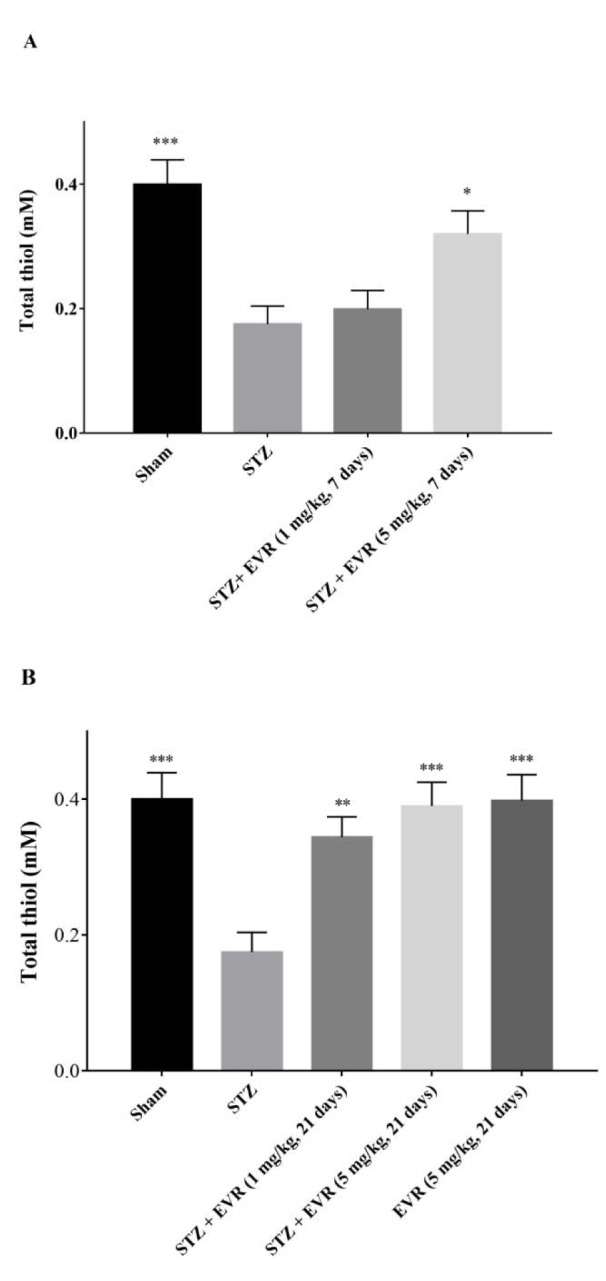
Effects of 7 days (A, on days 21-28 post-STZ infusion, after establishment of cognitive dysfunction) or 21 days (B, from day 0 which refers to the day of surgery and icv-STZ infusion) administration of EVR on total thiol level in the hippocampus of STZ-induced AD rats. Values are means ± SEM. *p<0.05, **p < 0.01 and ***p < 0.001 *vs.* STZ group.

The total thiol concentration was measured to evaluate the non-enzymatic defense potential against the oxidative stress. As shown in Figure 7(Fig. 7), following AD induction by icv-STZ, the hippocampal thiol concentrations were significantly decreased, as compared to the sham group (p < 0.001). As illustrated in Figure 7A(Fig. 7), treatment with EVR 5 mg/kg (p < 0.05) significantly restored the decreased sulfhydryl groups in the hippocampus, when compared with the STZ group.

Also, chronic administration of EVR (1 and 5 mg/kg) resulted in a significant increment of total thiol levels, as compared to the STZ group (p < 0.01, p < 0.001, respectively). The levels of thiol were not significantly (p > 0.05) altered by the administration of EVR (5 mg/kg for 21 days) alone, as shown by the absence of significant differences between the EVR group and the sham group (Figure 7B(Fig. 7)).

### Effect of EVR on activity of acetylcholinesterase

**Figure 8:**
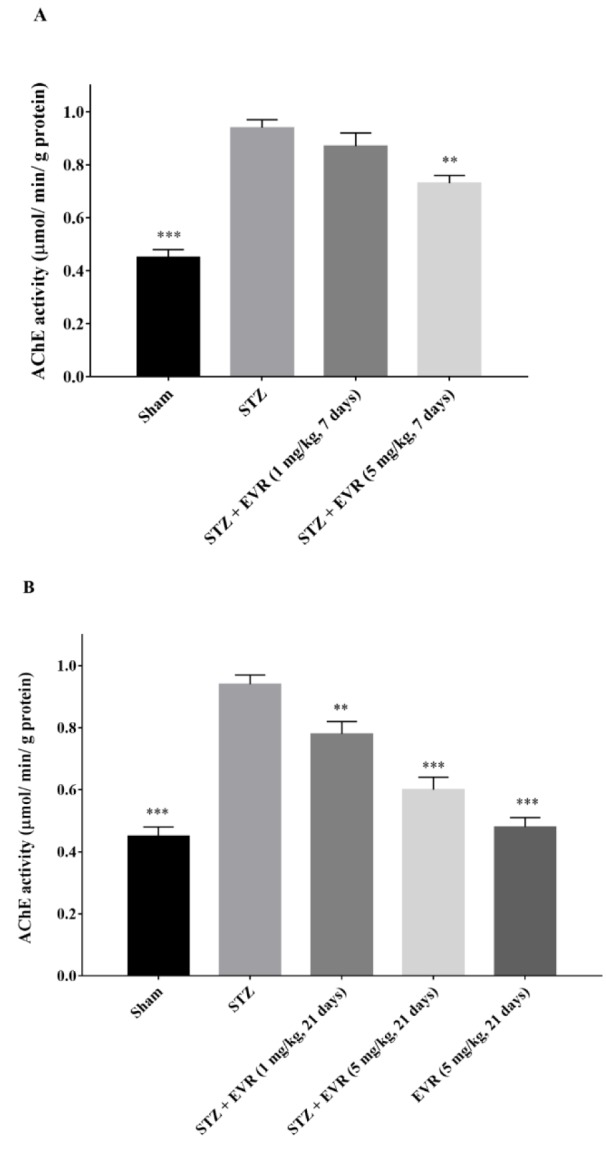
Effects of 7 days (A, on days 21-28 post-STZ infusion, after establishment of cognitive dysfunction) or 21 days (B, from day 0 which refers to the day of surgery and icv-STZ infusion) administration of EVR on acetylcholinesterase (AChE) activity in the hippocampus of STZ-induced AD rats. Values are means ± SEM. **p < 0.01, ***p < 0.001 *vs.* STZ group.

Figure 8A-B(Fig. 8) shows that there was a significant (p < 0.001) increase in the AChE activity in the hippocampus of STZ injected rats, as compared to the sham group. Treatment with 5 mg/kg EVR (for 7 days) significantly decreased the AChE activity *vs* the STZ group (p < 0.01) (Figure 8A(Fig. 8)). 

The increase in the activity of AChE was also significantly attenuated by the chronic administration of EVR (1 and 5 mg/kg) when compared to the STZ group (Figure 8B(Fig. 8), p < 0.01, p < 0.001, respectively).

### Effect of EVR on hippocampal cell loss

**Figure 9:**
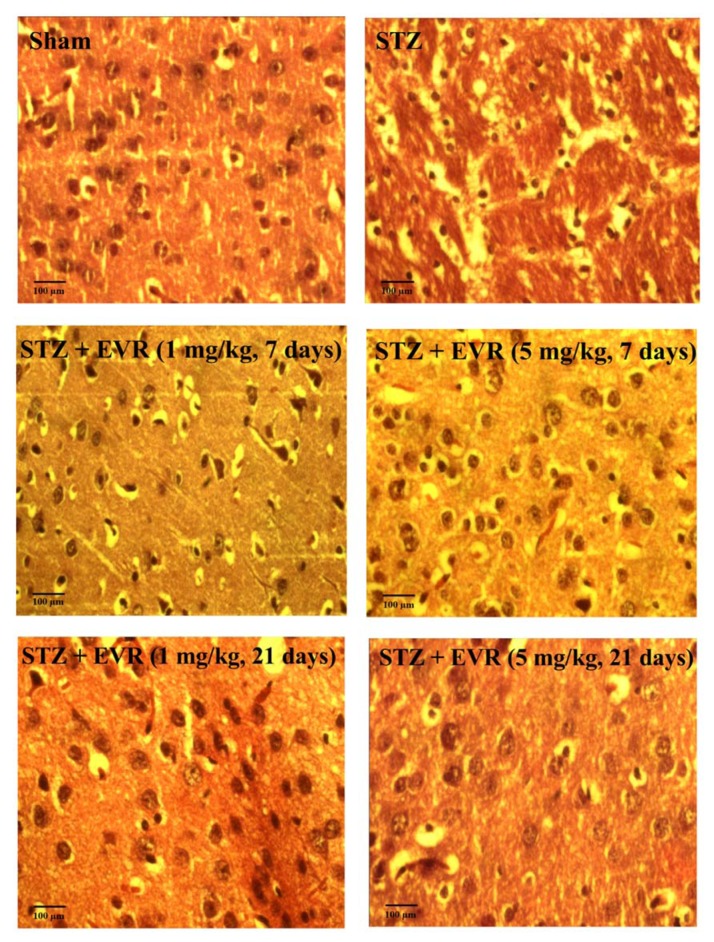
Effects of 7 days (on days 21-28 post-STZ infusion, after establishment of cognitive dysfunction) or 21 days (from day 0 which refers to the day of surgery and icv-STZ infusion) administration of EVR on cell density of degenerated cells in the hippocampal CA1 region of STZ-induced AD rats.

**Figure 10:**
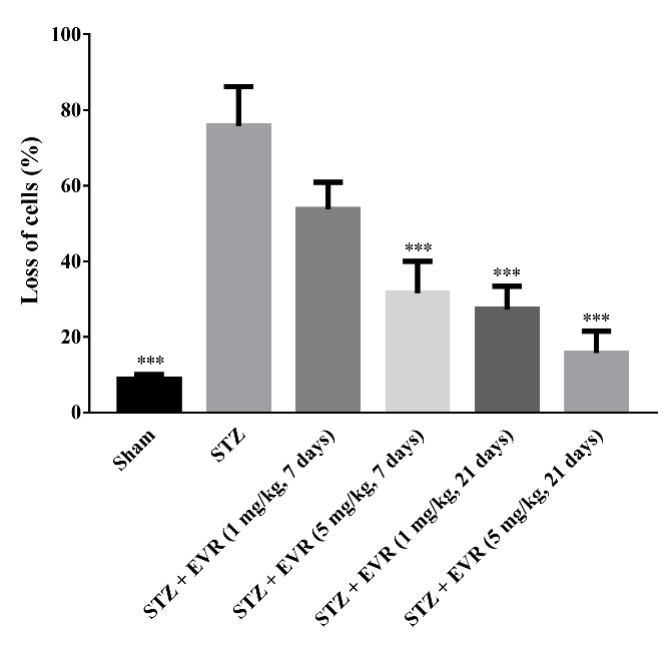
Effects of 7 days or 21 days administration of EVR on percentage of degenerated cells in the hippocampal CA1 region of STZ-induced AD rats

The representative H&E staining of the Cornu Ammonis (CA1) region of the hippocampus in different treatment groups has been shown in Figure 9(Fig. 9). The STZ group exhibited greater cell nuclei shrinkage than those of the EVR-treated rats. The STZ group revealed a large number of degenerated cells compared with the STZ-EVR group. Statistical analysis revealed significant neuronal cell loss in the hippocampal CA1 subfield of the AD animals compared with the sham group. Acute injection of EVR at a high dose (5 mg/kg) for 7 days or chronic administration for 21 days significantly attenuated hippocampal cell loss in comparison with the STZ AD rats (Figure 10(Fig. 10)). In addition, there was no significant difference in the CA1 neuronal cell loss between animals that received EVR (5 mg/kg) alone and the sham group (data not shown).

See also the Supplementary data.

## Discussion

The present study demonstrated that icv-STZ administration impaired learning and memory, increased oxidative stress, hippocampal cell loss, and induced cholinergic dysfunction in the rat hippocampus, which are consistent with previous studies (Ishrat et al., 2006[32]; Nazifi et al., 2018[53]; Nitsch and Hoyer, 1991[54]; Sharma and Gupta, 2001[71]; Shoham et al., 2007[73]). Our findings revealed that short-term treatment with a high dose of EVR (5 mg/kg for 7 days on days 21-28 post STZ injection when the learning and memory impairments were established) or chronic administration of low (1 mg/kg) or high (5 mg/kg) doses of EVR from day 0 (day of STZ icv injection) to day 20, were able to attenuate STZ-induced learning and memory deficits by inhibiting oxidative stress, amelioration of neuronal injury, and restoration of AChE activity in the hippocampus. The doses and time schedule of the administration of EVR were selected based on preliminary experiments, as well as, previous studies (Russo et al., 2016[65]; Sanchez-Fructuoso, 2008[68]; Yokomasu et al., 2009[90]). 

The passive avoidance test was performed for the assessment of learning and memory deficits in the rats. In this task, the cognitive ability of the animals was indicated by avoiding entry into the dark chamber due to the painful experience of the electric foot shock. STZ-administered rats showed reduction in retention latencies in passive avoidance behavior, demonstrating learning and memory impairments. These results are consistent with previous investigations that indicated cognitive impairment after icv-STZ in rats (Javed et al., 2012[34]; Mansouri et al., 2013[49]).

On the other hand, treatment with EVR significantly increased the latency time in two retention trials of the test, which means STZ-induced decreased retention latency was reversed by EVR. The MWM performed in this study is a well-validated behavioral model to evaluate spatial memory and learning in rodents (D'Hooge and De Deyn, 2001[23]). As such, a marked decline in latency during the trials (4 trials/day for 5 days) shows intact acquisition or learning ability. Furthermore, a normal retrieval memory was indicated by increased time spent in the target quadrant. STZ-injected rats showed a significant increase in the latency time and a decrease in time spent in the target quadrant, whereas improvement of spatial memory by the EVR treatment in STZ-injected rats was confirmed by shorter escape latencies and longer durations in the target quadrant, suggesting enhanced learning ability and memory consolidation by EVR. Our results were supported by recent reports in which EVR improved learning and memory in a mouse model of vascular dementia (Chen et al., 2016[17]) and depression (Russo et al., 2016[65]). 

It has been well documented that oxidative stress is an important factor in the pathogenesis of AD and cognitive deficits (Hamilton and Holscher, 2012[29]; Su et al., 2008[77]; Sultana and Butterfield, 2013[78]; Texel and Mattson, 2011[80]). It has been shown that icv-STZ administration caused oxidative stress via an increase in the MDA level and decrease in the glutathione level (Agrawal et al., 2009[2]; Kamat et al., 2016[37]), which were similar to our results. Our findings, along with a previous report, revealed an imbalance in the oxidative and antioxidative system parallel with cognitive dysfunction in STZ rats, indicated that learning and memory impairments are related to oxidative stress in this model (Saxena et al., 2008[70]). In the present study, treatment with EVR attenuated the STZ-induced increase in lipid peroxidation and decrease in the total thiol level, proposing that the protective effects of EVR against STZ might be partly related to increases in antioxidant defense. The mTOR signaling pathway is closely related to oxidative stress or antioxidant capacity. mTOR not only modulates oxidative stress, but is also affected by reactive oxygen species (ROS) (Zhao et al., 2017[92]). 

It has been shown that mTOR promotes oxidative stress-induced apoptosis of rat mesangial cells exposed to high glucose, while pretreatment of the cells with rapamycin, as an mTOR inhibitor, ameliorated oxidative stress and reduced the number of apoptotic cells (Lu et al., 2018[45]). Hyperactivation of mTORC1 eventually induces endoplasmic reticulum stress, mitochondrial oxidative stress and activation of apoptosis process (Wang et al., 2016[87]). Moreover, the higher activity of glutathione reductase has been reported in everolimus-treated mice (Kezic et al., 2013[38]). Pharmacologic inhibition of TORC1 signaling by rapamycin also restored the elevated levels of MDA and 4-hydroxyalkenals in a Drosophila model of Friedreich's Ataxia (Calap-Quintana et al., 2015[15]). Our results are also concordant with an earlier report, which revealed the protective effects of rapamycin on Aβ1-42-induced AD via its antioxidation function (Singh et al., 2017[74]). 

Furthermore, we showed that the administration of EVR reversed STZ-induced increase in the AChE activity in the hippocampus. It has been established that the cholinergic system has an essential role in the regulation of learning and memory and alters during aging and AD progression (Anand and Singh, 2013[4]; Lagarde et al., 2017[42]). AChE inhibitors, by inhibiting the hydrolysis of acetylcholine, enhance the cholinergic transmission and temporarily decrease the AD-induced cognitive deficits (Darreh-Shori et al., 2004[21]). In line with our finding, previous studies also showed increased AChE activity and concurrent memory impairments in STZ-induced AD rats (Agrawal et al., 2009[2]; Gutierres et al., 2014[28]; Saxena et al., 2008[70]; Sharma et al., 2012[72]; Tiwari et al., 2009[81]). 

Moreover, it has been shown that activation of autophagy following the administration of rapamycin restored the elevated AChE activity in AD induced by Aβ1-42 (Singh et al., 2017[74]). mTOR signaling is a pivotal regulator for neural cell metabolism, growth and proliferation, redox status, and autophagy (Maiese, 2014[46]). mTOR complexes (mTORC1 and mTORC2) are directly and indirectly involved in the regulation of autophagy (Abada and Elazar, 2014[1]; Kim et al., 2011[40]). Autophagy is defined as a major degradation pathway in eukaryotic cells that is vital for eliminating damaged organelles and macromolecules from the cytoplasm and reprocessing amino acids in periods of starvation (Mizushima et al., 2008[51]). In fact, autophagy controls cellular stress conditions such as oxidative load, and improves neuronal survival (Damme et al., 2015[19]). Thus, the imperfect autophagy leads to accumulation of damaged organelles and toxic protein aggregates that contribute to neuronal death and pathogenesis of several neurodegenerative diseases including AD (Li et al., 2010[44]). It has been revealed that autophagy may be a part of pro-survival (PI3K/Akt/ mTOR/CREB) signaling, and the activation of autophagy results in the restoration of oxidative defense mechanisms and neuronal damages as well as conserving the integrity of synapse and neurotransmission in the rat model of AD (Singh et al., 2017[74]). 

Besides, the continuous activation of the neuronal PI3K/Akt/mTOR pathway has been related with the induction of insulin resistance in the AD brain. It has been indicated that the effect of glucose on the augmentation of memory can be regulated via mTOR signaling (Dash et al., 2006[22]) and in the insulin resistance condition the mTOR pathway is dysregulated (Dann et al., 2007[20]; Zick, 2005[94]). It was also confirmed that the hyperactivation of mTOR causes the inhibition of insulin receptor signaling (Gupta and Dey, 2012[27]). Therefore, there is a molecular link between AD pathology and insulin resistance via mTOR hyperactivation (Barone et al., 2016[6]; Caccamo et al., 2010[12]; Perluigi et al., 2014[58]; Tramutola et al., 2015[82]). mTOR hyperactivation, by blocking autophagy, is involved in the pathologic accumulation of tau and Aβ proteins and associated neurodegeneration (Caccamo et al., 2013[12]; Khurana et al., 2006[39]; Tang et al., 2015[79]). 

mTOR is also essential for synaptic plasticity and memory formation (Braak and Braak, 1991[10]), and mTOR signaling hyperactivity has detrimental effects on learning and memory functions (Bolduc et al., 2008[9]). On the other hand, mTOR inhibition could improve learning and memory functions, reduce tau pathology (Caccamo et al., 2010[13]), increase autophagy and decrease the amyloid β level in animal models of AD (Spilman et al., 2010[75]). mTOR inhibition can also increase lifespan and delay age-related cognitive impairments (Johnson et al., 2013[35]; Maiese et al., 2013[47]). 

The beneficial effects of EVR seen in this study are in accordance with some previous results from rapamycin, which is able to ameliorate age-dependent learning and memory impairments (Majumder et al., 2012[48]) and reduce cognitive deficits related to neurological disorders such as AD (Spilman et al., 2010[75]) and status epilepticus (Brewster et al., 2013[11]). It has been previously shown that EVR acted as a protector against cognitive decline by increasing neurogenesis in an animal model of depression (Russo et al., 2016[65]). Therefore, mTOR inhibitors might be considered as modifying agents for AD. 

In conclusion, our study demonstrated, for the first time, that chronic or acute administration of EVR protected against cognitive impairments and deleterious alterations in oxidative status and AChE activity in the hippocampus of the STZ-AD rats. It presents new opportunities for drug development against AD. 

## Acknowledgement

This study was financially supported by the Vice Chancellery for Research and Technology, Mashhad University of Medical Sciences (under grant number: 941697). The authors are grateful for this financial support.

## Conflict of interest

The authors declare that they have no conflict of interest.

## Supplementary Material

Supplementary data
