# Supplementary material for: Everolimus, a mammalian target of rapamycin inhibitor, ameliorated streptozotocin-induced learning and memory deficits via neurochemical alterations in male rats
Source: EXCLI J. 2018 Oct 29;17:999–1017. doi: 10.17179/excli2018-1626 (PMC6295637; doi:10.17179/excli2018-1626)
Supplement: Supplementary data [file EXCLI-17-999-s-001.pdf]

EXCLI Journal 2018;17:999-1017 – ISSN 1611-2156
Received: August 16, 2018, accepted: October 05, 2018, published: October 29, 2018

Supplementary data to:

EVEROLIMUS, A MAMMALIAN TARGET OF RAPAMYCIN INHIBITOR, AMELIORATED STREPTOZOTOCIN-INDUCED LEARNING AND MEMORY DEFICITS VIA NEUROCHEMICAL ALTERATIONS IN MALE RATS

Sahar Fanoudi[a,b], Mahmoud Hosseini[c], Mohaddeseh Sadat Alavi[a,b], Mohammad Taher Boroushaki[a,b], Azar Hosseini[b], Hamid R. Sadeghnia[a-c*]

[a] Department of Pharmacology, Faculty of Medicine, Mashhad University of Medical Sciences, Mashhad, Iran
[b] Pharmacological Research Center of Medicinal Plants, Mashhad University of Medical Sciences, Mashhad, Iran
[c] Division of Neurocognitive Sciences, Psychiatry and Behavioral Sciences Research Center, Mashhad University of Medical Sciences, Mashhad, Iran

* **Corresponding author:** Hamid R. Sadeghnia, Department of Pharmacology, Pharmacological Research Center of Medicinal Plants, Division of Neurocognitive Sciences, Psychiatry and Behavioral Sciences Research Center, Faculty of Medicine, Mashhad University of Medical Sciences, Mashhad, PO Box 99199-91766, Iran, Tel: +98 513 8828566, Fax: +98 513 8828567, E-mail: sadeghniahr@mums.ac.ir

http://dx.doi.org/10.17179/excli2018-1626

This is an Open Access article distributed under the terms of the Creative Commons Attribution License (http://creativecommons.org/licenses/by/4.0/).

EXCLI Journal 2018;17:999-1017 – ISSN 1611-2156
Received: August 16, 2018, accepted: October 05, 2018, published: October 29, 2018

Supplementary Table 1 (to Figure 2): Effects of 7 days or 21 days administration of Everolimus (EVR) on passive avoidance memory of streptozotocin (STZ)-induced Alzheimer's disease (AD) rats.

Group	Animal number	Before shock (sec)	3 h after shock (sec)	24 h after shock (sec)
**Sham**	1	5	200	220
	2	8	280	150
	3	40	300	280
	4	5	155	140
	5	4	300	300
	6	14	300	295
	7	5	222	235
	8	4	245	260
	9	18	212	192
	10	2	287	278
	Mean ± SEM	10.5 ± 3.64	250.1 ± 16.17	235 ±18.45
**STZ**	1	4	5	8
	2	4	6	5
	3	6	60	45
	4	44	12	5
	5	45	100	85
	6	3	75	60
	7	6	100	49
	8	4	45	38
	9	5	2	4
	10	10	3	6
	11	5	4	2
	12	59	85	73
	Mean ± SEM	16.25 ± 5.87	41.41 ± 11.71	31.66 ± 8.77
**STZ + EVR (1 mg/kg, 7 days)**	1	7	4	39
	2	45	250	280
	3	30	5	57
	4	50	130	150
	5	30	150	3
	6	49	294	296
	7	21	43	14
	8	38	71	57
	9	44	208	108
	10	10	21	53
	Mean ± SEM	32.4 ± 4.94	117.6 ± 33.44	105.7 ± 33.24
**STZ + EVR (5 mg/kg, 7 days)**	1	59	300	300
	2	5	300	180
	3	5	300	300
	4	7	300	300
	5	4	5	10
	6	4	64	29
	7	58	300	296
	8	27	300	292
	9	4	294	252
	Mean ± SEM	19.22 ± 7.81	240.33 ± 39.21	217.66 ± 39.67
**STZ + EVR (1 mg/kg, 21 days)**	1	8	300	250
	2	5	100	86
	3	5	253	210
	4	5	100	85
	5	14	157	113
	6	5	253	210
	7	12	160	88
	8	3	273	190
	9	7	87	110

EXCLI Journal 2018;17:999-1017 – ISSN 1611-2156
Received: August 16, 2018, accepted: October 05, 2018, published: October 29, 2018

	10	6	253	245
	Mean ± SEM	7 ± 1.09	193.6 ± 25.72	158.7 ± 21.65
**STZ + EVR (5 mg/kg, 21 days)**	1	5	300	285
	2	4	10	150
	3	14	300	300
	4	6	300	300
	5	6	250	198
	6	11	253	267
	7	6	296	271
	8	11	16	125
	9	4	293	300
	10	3	300	269
	Mean ± SEM	7.00 ± 1.16	231.8 ± 36.97	246.5 ± 20.55
**EVR (5 mg/kg, 21 days)**	1	35	250	300
	2	38	300	150
	3	10	60	72
	4	5	263	211
	5	8	230	242
	6	7	242	234
	7	33	300	250
	8	9	243	72
	9	25	10	46
	10	48	282	253
	Mean ± SEM	21.8 ± 5	218 ± 31.65	183 ± 28.76

EXCLI Journal 2018;17:999-1017 – ISSN 1611-2156
Received: August 16, 2018, accepted: October 05, 2018, published: October 29, 2018

Supplementary Table 2 (to Figure 3): Effects of 7 days or 21 days administration of EVR on escape latency of STZ-induced AD rats in Morris water maze (MWM) task.

Group name	Animal num-ber	Trials	Day 1 (sec)	Day 2 (sec)	Day 3 (sec)	Day 4 (sec)	Day 5 (sec)
**Sham**	1	1	59.77	24.8	25.63	16.63	17.97
		2	59.73	29.67	4.83	9.97	7.13
		3	59.73	6.67	1.73	22.43	12.03
		4	30.90	22.5	21	3.97	15.40
	2	1	59.77	12.53	3	2.6	15.77
		2	29.4	18.77	5.8	18.03	7.57
		3	5	10	6.73	0.87	5.67
		4	29.07	11.7	2.03	23.77	5.1
	3	1	52.8	11	7.6	3.07	20.25
		2	7.17	6.5	10.6	30.17	2.1
		3	15.53	5.03	4.23	4.2	14.23
		4	12.83	10.6	2.07	7.9	10.77
	4	1	59.17	8.1	58.9	25.5	24.27
		2	59.73	5.5	3.63	13.3	19.57
		3	59.73	34.07	4.6	19.97	3.47
		4	59.73	18.17	2.7	3.97	3.57
	5	1	59.77	25.4	14.4	4.7	1.73
		2	32.97	15.1	17.73	23.3	5.6
		3	11.03	27.87	2.03	2.87	2.37
		4	38.6	8.57	8.93	13.07	12.2
	6	1	59.73	19.8	14.2	9	4.8
		2	42.03	6.83	1.77	6.1	8.07
		3	18.3	33.9	16.4	3.3	14
		4	19.27	10.98	1.33	14	5.8
	7	1	59.03	8	2.53	9	5.27
		2	25.67	24.53	4.77	1.27	1.73
		3	36.9	18.77	18.17	3.07	3.53
		4	59.77	21.8	22	1.83	1.83
	8	1	45.47	9.93	11.33	14	10.87
		2	40.8	59.73	4.97	12.37	5.4
		3	59.73	35.2	1.43	3.47	16.7
		4	48	13.07	25.07	20.73	3.13
	9	1	59.73	25.59	29.39	17.62	21.47
		2	43.56	31.03	8.31	16.78	3.75
		3	4.17	5.15	1.33	1.76	12.55
		4	35.72	7.46	3.36	6.26	1.88
	10	1	59.77	34.62	26.63	20.81	12.12
		2	53.92	22.52	10.08	1.4	8.26
		3	29.5	6.77	1.45	3.39	4.11
		4	39.5	10.60	6.86	19.04	7.79
		Mean ± SEM	41.08 ± 2.95	17.97 ± 1.85	10.49 ± 1.79	10.88 ± 1.31	8.99 ± 0.99
**STZ**	1	1	59.77	59.77	15.57	19.4	40.77
		2	59.77	38.73	32.17	17.83	33.43
		3	59.77	19.63	15.6	16.57	52.33
		4	59.77	48.8	29.57	27.17	13.23
	2	1	59.73	59.73	15.6	14.9	25.77
		2	59.77	59.77	44.47	30	32.27
		3	9.33	13.17	13.43	59.77	17.97
		4	59.77	30.87	11.43	17.13	17.4
	3	1	59.73	59.77	59.77	21.03	28
		2	19.73	13.63	19.7	34.63	15.2
		3	30.27	56.23	11.4	16.8	16.97

		4	10.37	26.83	14.77	36	16.13
	4	1	26.03	23.87	16.1	15.3	38.67
		2	59.73	28.2	13.83	16.17	10.67
		3	35.53	8.4	27.2	39.7	14.97
		4	16.3	37.87	41	15.57	13.6
	5	1	59.77	36.97	34.4	20.3	17.4
		2	16.37	38.5	14	17.8	17.77
		3	6.47	59.77	15	16.37	14.5
		4	20.4	27.2	14.2	30	13.3
	6	1	59.77	59.77	17.5	17.5	24.97
		2	59.73	11.8	14.03	32.3	14.73
		3	33.23	33.93	53.8	15.4	14.53
		4	59.77	59.77	59.77	16.3	25.9
	7	1	59.73	59.77	32.3	48.07	59.77
		2	59.77	59.77	59.77	59.73	53.93
		3	59.73	59.77	59.77	48.27	13.63
		4	59.77	59.77	59.77	59.77	59.77
	8	1	46.37	14.5	15	13.2	59.77
		2	59.77	14.9	13	19.7	16.5
		3	20.8	13.93	32.8	18.97	15.3
		4	22.83	19.17	19.77	14.67	14.43
	9	1	59.77	59.77	25.37	17.43	10.8
		2	59.77	49.03	11	18.47	42.97
		3	59.77	27.17	12	16	27.17
		4	59.73	32.43	42.17	18.53	14.17
	10	1	59.77	15.8	59.73	39.9	15.8
		2	59.77	26.77	59.77	15.2	9.2
		3	59.73	59.77	12.13	15	14.8
		4	59.77	31.07	10.43	16.03	7.27
	11	1	59.77	59.73	59.77	59.77	55.2
		2	59.77	19.87	26.4	20.8	46.27
		3	59.77	59.77	59.73	59.77	59.73
		4	59.73	59.77	59.73	59.77	59.77
	12	1	59.73	59.77	40.97	32.1	26.27
		2	59.77	32.1	43.6	20.8	23.6
		3	59.73	17.83	29.2	13.37	23.8
		4	59.77	25.2	12.2	11	10
		Mean ± SEM	48.87 ± 2.61	38.55 ± 2.67	30.43 ± 2.67	26.67 ± 2.23	26.46 ± 2.39
**STZ + EVR (1 mg/kg, 7 days)**	1	1	59.77	17.67	42.27	38.33	16.07
		2	59.77	59.73	9	5.57	15
		3	33.4	18.3	10.13	14.43	18.17
		4	59.73	15.5	51.77	15.43	15.47
	2	1	59.73	59.77	6.3	13.1	22.33
		2	33.4	20.22	54.4	59.77	43.5
		3	59.77	59.43	59.73	32.6	17.97
		4	20.1	59.77	15	59.77	40.43
	3	1	59.77	15.2	51.7	22.97	31.87
		2	59.73	16.63	9.17	12.17	25.36
		3	22.17	41.6	24	13.25	18.32
		4	59.73	48.63	4.73	12.32	15.57
	4	1	59.77	54.33	40	32.9	17.77
		2	59.77	10.97	23	22.63	18.37
		3	25.3	14.35	20.13	37.9	20.32
		4	59.77	55.5	54	26.97	18.43
	5	1	59.77	32	35	41.5	20.12
		2	59.77	59.77	23.97	16.13	18.2
		3	37.8	12	7.03	13.2	25.36
		4	59.77	35	39.83	18.77	37.27

	6	1	59.77	59.77	36.53	37.85	43.44
		2	53.67	39.98	39.46	35.95	15.29
		3	22.51	34.8	4.45	6.29	23.63
		4	57.57	49.1	32.17	19.82	18.02
	7	1	45.65	39.64	37.51	51.54	20.38
		2	38.18	57.53	3.75	40.88	15.19
		3	35.03	56.96	13.63	31.25	19.17
		4	55.80	15.81	10.90	15.80	23.22
	8	1	59.77	59.73	53.98	49.98	35.30
		2	56.02	27.65	3.63	26.38	15.83
		3	40.6	7.11	47.53	14.22	25.41
		4	59.31	33.94	45.76	20.08	19.69
	9	1	50.79	46.57	31.99	20.44	32.83
		2	59.77	13.76	46.40	19.46	23.64
		3	18.09	6.01	49.49	15.56	24.62
		4	56.42	30.45	13.02	4.34	17.55
	10	1	59.77	59.77	59.77	57.00	33.30
		2	50.56	43.44	5.20	5.70	3.50
		3	25.1	7.45	24.73	23.75	23.28
		4	16.11	15.21	20.62	13.30	22.51
		Mean ± SEM	48.23 ± 2.38	35.27 ± 3.04	29.04 ± 2.92	25.48 ± 2.43	22.79 ± 1.34
**STZ + EVR (5 mg/kg, 7 days)**	1	1	35.47	3.33	19.73	18.97	54.8
		2	59.77	14.3	2.8	2.17	7
		3	37.17	3.2	46.5	2.77	14.07
		4	59.77	4.3	14.93	5.23	16.50
	2	1	59.77	59.77	58.83	6.1	5.73
		2	50.93	11.5	14.07	11.23	18.01
		3	59.77	18.67	11.53	0.8	7.33
		4	14.57	19.97	39.77	5.17	29.27
	3	1	42.03	38.9	19.23	1.77	12.9
		2	4.47	15.7	18.83	31	11.63
		3	42.17	23.97	11.47	10.1	9.8
		4	59.73	18.57	8.83	21.83	4.47
	4	1	59.73	52.17	14.63	59.73	44.8
		2	23.77	23.87	16.77	5.73	5.33
		3	59.77	32.73	8.03	45.47	2.9
		4	59.77	2.27	6.13	10.1	5.98
	5	1	59.77	59.77	10.83	9.8	6.3
		2	1.23	6.57	11	9.03	15.2
		3	59.77	53.8	14.57	5.07	2.07
		4	4.7	15.6	6.47	11.2	11.5
	6	1	59.77	43.54	26.57	6.12	16.61
		2	59.77	19.11	28.01	7.80	13.83
		3	45.06	19.00	16.52	16.36	3.58
		4	21.03	2.3	37.22	17.96	6.53
	7	1	54.00	43.9	40.85	24.24	2.74
		2	59.77	1.61	23.75	3.49	15.34
		3	59.77	27.55	6.80	17.47	10.59
		4	54.65	3.1	36.02	3.08	1.83
	8	1	59.77	55.95	31.16	52.44	47.33
		2	27.61	15.99	8.00	8.11	13.16
		3	20.97	27.7	3.19	1.911	31.97
		4	58.38	15.3	1.97	6.37	6.13
	9	1	50.19	59.77	13.58	13.95	19.79
		2	3.6	28.29	2.18	34.10	2.64
		3	12.63	11.05	12.05	14.86	10.77
		4	31.47	13.27	3.1	2.84	1.6

		Mean ± SEM	42.57 ± 3.40	24.06 ± 3.09	17.94 ± 2.30	14.01 ± 2.39	13.61 ± 2.16
**STZ + EVR (1 mg/kg, 21 days)**	1	1	47	21.3	5	15.67	7.37
		2	59.77	2.93	9.77	23.93	2.37
		3	37.13	10.07	3.93	9.43	0.87
		4	14.73	4.1	18.07	13.27	3.2
	2	1	59.77	58.2	18.77	6.37	24.27
		2	59.77	2.07	2.07	4.77	6.93
		3	34.13	8.5	11.2	6.67	20.07
		4	59.73	13.27	5.4	8.73	1.2
	3	1	59.73	56.13	25.6	13.87	3.53
		2	59.77	9.57	41.1	3.77	4.6
		3	59.77	3.37	2.87	1.17	8.13
		4	3.3	6.4	6.63	4.03	1.97
	4	1	59.77	54.57	1.6	14.7	10.4
		2	35.07	26	5.07	10.73	5.17
		3	55.03	4.27	4.7	6.97	3.2
		4	12.83	2.63	4.5	1.5	23.1
	5	1	59.73	59.77	3.03	4.83	6.6
		2	59.77	40.53	31.47	4.27	7.67
		3	38.87	19.23	59.77	59.73	23.63
		4	57.2	2.07	59.77	42.6	4.33
	6	1	56.08	59.77	24.22	21.12	15.21
		2	41.96	22.90	13.22	11.69	15.54
		3	23.76	37.43	2.00	2.03	5.03
		4	33.790	11.58	2.76	38.54	2.99
	7	1	59.77	57.24	13.27	2.03	16.2
		2	22.45	15.95	6.8	14.84	23.75
		3	57.46	10.78	13.34	5.39	1.49
		4	47.33	30.56	3.30	10.65	2.30
	8	1	57.98	55.32	28.06	2.87	1.77
		2	7.99	2.65	19.20	35.47	2.80
		3	34.6	2.3	27.63	1.86285	4.20
		4	59.77	39.10	8.66	1.72	8.26
	9	1	59.48	16.03	26.81	20.60	16.16
		2	59.03	5.63	7.55	9.93	4.23
		3	37.79	3.7	14.14	3.27	11.49
		4	59.77	8.42	59.77	2.57	2.10
	10	1	57.60	20.49	22.83	6.55	15.31
		2	34.36	2.56	25.18	24.26	5.92
		3	59.77	7.00	1.87	32.62	13.76
		4	59.77	2.84	2.12	18.71	2.15
		Mean ± SEM	46.58 ± 2.67	20.43 ± 3.17	16.07 ± 2.54	13.09 ± 2.08	8.48 ± 1.14
**STZ + EVR (5 mg/kg, 21 days)**	1	1	59.77	15.43	16.4	4.07	12.67
		2	9.5	4.97	7.97	2.03	4.23
		3	36.03	33.8	2.03	5.57	3.6
		4	59.77	28.7	11.8	10.43	5.07
	2	1	48.53	37.46	22.53	13.1	18.03
		2	37.8	11.33	18.47	1.4	4.77
		3	59.77	39.77	23.63	4.53	0.6
		4	59.77	16.57	8	16.87	27.2
	3	1	30.23	3.67	4.33	2.53	25
		2	26.37	30.77	7.4	12.57	3.3
		3	25.67	17.33	12.2	1.13	2.4
		4	49.5	1.33	14.7	8.63	5.17
	4	1	59.77	28.73	19.73	36.4	15.43
		2	59.77	40.77	21.6	28	29.77
		3	59.77	40.1	23.66	39.77	11.8

		4	46.9	29.73	17.46	26	1.23
	5	1	48.83	38.23	31.32	17.59	16.15
		2	59.77	19.71	20.95	30.42	3.94
		3	31.79	23.81	22.45	9.11	2.03
		4	59.77	26.10	12.85	1.70	8.83
	6	1	59.77	26.77	5.91	36.56	32.16
		2	24.71	30.94	22.98	27.34	24.03
		3	43.26	6.10	7.18	1.77	2.9
		4	22.06	32.86	12.02	14.29	8.72
	7	1	59.77	10.91	26.62	29.90	30.33
		2	21.76	20.68	10.62	36.34	11.04
		3	59.77	22.55	2.14	1.64	16.61
		4	49.25	5.47	9.95	7.89	3.95
	8	1	39.01	26.34	13.94	7.58	12.56
		2	49.10	39.73	11.37	32.35	2.30
		3	53.51	12.98	13.76	8.06	7.21
		4	59.77	39.57	10.25	11.18	8.01
	9	1	59.77	46.71	5.15	7.56	12.9
		2	48.30	9.5	17.59	5.31	4.93
		3	37.82	5.49	13.54	1.87	12.06
		4	58.97	22.37	11.00	6.08	11.96
	10	1	45.89	47.59	21.39	12.44	14.07
		2	38.00	11.39	13.56	5.94	7.29
		3	27.02	3.73	12.38	2.89	2.3
		4	26.57	41.04	18.81	7.51	2.04
		Mean ± SEM	45.33 ± 2.29	23.77 ± 2.14	14.49 ± 1.1	13.41 ± 1.89	10.71 ± 1.39
**EVR (5 mg/kg, 21 days)**	1	1	59.77	23.43	29.93	4.13	27.07
		2	45.83	22.77	10.93	11.17	7.3
		3	34.5	17.77	16.93	10	4.27
		4	5.37	17.17	3.47	5.93	3.1
	2	1	59.77	13.1	15.07	13.87	2.87
		2	12	26.27	4.4	29.77	8.63
		3	59.73	10.1	1.47	6.4	1.8
		4	59.77	11.65	9.57	1.5	2.6
	3	1	59.77	29.77	22.07	19.03	9.43
		2	59.77	9.27	25.4	2.5	1.17
		3	59.77	4.17	22.57	5.13	1.7
		4	59.73	1.07	8.37	3.3	1.6
	4	1	59.73	24.83	11.40	17.24	10.05
		2	29.34	17.15	6.79	2.84	1.41
		3	56.31	11.05	2.55	24.77	18.77
		4	52.08	2.97	16.76	3.20	23.78
	5	1	59.77	14.81	8.38	3.01	5.08
		2	39.95	13.03	8.54	18.26	6.12
		3	49.22	9.88	2.47	2.93	1.26
		4	59.84	11.85	10.14	16.35	1.36
	6	1	59.77	26.64	36.22	5.4	2.29
		2	8.50	33.72	16.35	4.16	3.81
		3	19.36	26.64	16.68	1.74	7.93
		4	59.77	15.37	15.81	14.16	5.81
	7	1	54.91	4.65	5.49	13.50	6.02
		2	59.77	11.68	6.36	14.64	1.74
		3	20.71	19.48	14.52	18.96	4.14
		4	31.43	32.58	25.83	6.49	9.45
	8	1	59.77	21.09	9.65	13.04	2.94
		2	25.94	27.21	30.75	2.548	13.58
		3	46.34	19.04	5.07	2.77	4.44
		4	51.82	17.48	34.36	3.98	3.84

	9	1	59.77	17.55	11.84	15.15	15.29
		2	52.10	10.55	18.67	10.02	5.12
		3	51.55	3.49	23.01	3.25	4.55
		4	59.77	5.00	20.16	1.98	1.44
	10	1	59.77	17.47	10.10	13.05	15.56
		2	42.55	10.78	5.37	15.30	9.29
		3	59.77	4.40	14.25	10.48	2.57
		4	44.85	4.73	10.39	10.92	4.73
		Mean ± SEM	47.76 ± 2.58	15.54 ± 1.37	14.20 ± 1.43	9.57 ± 1.11	6.59 ± 0.97

EXCLI Journal 2018;17:999-1017 – ISSN 1611-2156
Received: August 16, 2018, accepted: October 05, 2018, published: October 29, 2018

Supplementary Table 3 (to Figure 4): Effects of 7 days or 21 days administration of EVR on time spent in target quadrant of STZ-induced AD rats in Morris water maze (MWM) task.

Group name	Animal number	trials	Time in target quadrant (sec)
Sham	1	1	29.93
		2	30.50
		3	25.73
		4	24.17
	2	1	24.27
		2	23.30
		3	20.03
		4	24.96
	3	1	25.67
		2	21.10
		3	30.43
		4	23.23
	4	1	34.23
		2	30.73
		3	28.63
		4	22.47
	5	1	28.10
		2	24.60
		3	20.63
		4	23.35
	6	1	32.97
		2	30.47
		3	19.63
		4	29.57
	7	1	28.50
		2	22.77
		3	28.27
		4	25.97
	8	1	21.93
		2	25.47
		3	26.00
		4	30.30
	9	1	24.58
		2	29.33
		3	23.30
		4	23.50
	10	1	26.61
		2	32.12
		3	20.57
		4	29.44
		Mean ± SEM	26.18 ± 0.60
STZ	1	1	17.23
		2	20.83
		3	15.90
		4	18.23
	2	1	21.80
		2	25.77
		3	22.87
		4	10.60
	3	1	21.57
		2	18.93
		3	17.20
		4	11.23
	4	1	19.83
		2	24.63

EXCLI Journal 2018;17:999-1017 – ISSN 1611-2156
Received: August 16, 2018, accepted: October 05, 2018, published: October 29, 2018

		3	16.23
		4	17.33
	5	1	20.73
		2	16.73
		3	11.20
		4	10.50
	6	1	16.70
		2	20.40
		3	21.57
		4	10.90
	7	1	11.43
		2	15.50
		3	18.00
		4	15.50
	8	1	26.40
		2	18.27
		3	25.53
		4	21.30
	9	1	25.67
		2	16.77
		3	14.57
		4	17.97
	10	1	14.67
		2	24.83
		3	12.60
		4	19.33
	11	1	18.03
		2	22.20
		3	17.27
		4	17.97
	12	1	24.09
		2	14.77
		3	19.14
		4	14.95
		Mean ± SEM	18.24 ± 0.62
**STZ + EVR (1 mg/kg, 7 days)**	1	1	10.60
		2	24.63
		3	26.40
		4	15.53
	2	1	12.07
		2	17.80
		3	24.27
		4	21.03
	3	1	25.90
		2	20.33
		3	19.57
		4	13.70
	4	1	27.23
		2	24.10
		3	24.77
		4	22.13
	5	1	10.50
		2	25.60
		3	16.70
		4	18.40
	6	1	24.37
		2	23.74
		3	18.96
		4	23.22

EXCLI Journal 2018;17:999-1017 – ISSN 1611-2156
Received: August 16, 2018, accepted: October 05, 2018, published: October 29, 2018

	7	1	16.02
		2	13.39
		3	17.98
		4	30.40
	8	1	19.27
		2	21.35
		3	29.86
		4	19.15
	9	1	24.76
		2	10.01
		3	16.46
		4	17.67
	10	1	19.17
		2	16.68
		3	12.48
		4	26.21
		Mean ± SEM	20.06 ± 0.85
**STZ + EVR (5 mg/kg, 7 days)**	1	1	25.87
		2	21.37
		3	30.73
		4	21.27
	2	1	23.23
		2	21.03
		3	19.63
		4	24.40
	3	1	25.23
		2	25.33
		3	19.73
		4	23.47
	4	1	25.83
		2	21.33
		3	19.33
		4	20.33
	5	1	20.30
		2	35.03
		3	35.20
		4	32.23
	6	1	24.60
		2	28.23
		3	26.37
		4	22.14
	7	1	21.50
		2	16.17
		3	23.23
		4	20.09
	8	1	26.26
		2	38.34
		3	28.78
		4	26.14
	9	1	21.64
		2	20.23
		3	27.28
		4	21.63
		Mean ± SEM	24.54 ± 0.82
**STZ + EVR (1 mg/kg, 21 days)**	1	1	20.70
		2	24.27
		3	25.67
		4	20.67
	2	1	27.73

		2	25.47
		3	25.77
		4	24.00
	3	1	29.60
		2	20.57
		3	26.90
		4	25.87
	4	1	30.20
		2	24.77
		3	30.73
		4	23.33
	5	1	25.90
		2	29.50
		3	23.77
		4	24.70
	6	1	25.20
		2	24.63
		3	26.79
		4	23.47
	7	1	29.59
		2	27.96
		3	28.59
		4	25.99
	8	1	22.39
		2	28.27
		3	26.13
		4	29.95
	9	1	22.83
		2	23.87
		3	18.82
		4	24.78
	10	1	29.26
		2	26.11
		3	25.05
		4	20.34
		Mean ± SEM	25.5 ± 0.47
**STZ + EVR (5 mg/kg, 21 days)**	1	1	24.30
		2	20.60
		3	27.53
		4	24.73
	2	1	30.97
		2	24.67
		3	24.13
		4	20.63
	3	1	28.83
		2	31.00
		3	29.10
		4	30.60
	4	1	20.90
		2	23.33
		3	26.20
		4	25.60
	5	1	21.56
		2	30.48
		3	24.46
		4	26.13
	6	1	30.02
		2	22.69
		3	23.84

EXCLI Journal 2018;17:999-1017 – ISSN 1611-2156
Received: August 16, 2018, accepted: October 05, 2018, published: October 29, 2018

		4	25.55
	7	1	22.35
		2	27.01
		3	28.82
		4	21.29
	8	1	22.16
		2	28.28
		3	16.92
		4	24.59
	9	1	29.62
		2	30.78
		3	28.97
		4	27.96
	10	1	27.98
		2	25.37
		3	23.35
		4	29.36
		Mean ± SEM	25.82 ± 0.55
**EVR (5 mg/kg, 21 days)**	1	1	25.37
		2	19.57
		3	26.90
		4	24.73
	2	1	25.40
		2	21.70
		3	30.93
		4	26.10
	3	1	23.30
		2	23.07
		3	34.33
		4	23.53
	4	1	19.37
		2	24.99
		3	25.96
		4	29.69
	5	1	20.41
		2	28.47
		3	25.59
		4	27.03
	6	1	33.19
		2	16.64
		3	30.21
		4	25.50
	7	1	31.63
		2	26.25
		3	25.49
		4	24.86
	8	1	27.84
		2	21.51
		3	19.47
		4	28.32
	9	1	20.75
		2	22.58
		3	22.14
		4	22.99
	10	1	26.70
		2	28.65
		3	28.86
		4	26.23
		Mean ± SEM	25.41 ± 0.62

EXCLI Journal 2018;17:999-1017 – ISSN 1611-2156
Received: August 16, 2018, accepted: October 05, 2018, published: October 29, 2018

Supplementary Table 4 (to Figure 5): Effects of 7 days or 21 days administration of EVR on swimming speed of STZ-induced AD rats in Morris water maze (MWM) task.

Group name	Animal number	Trials	Day 1 (cm/sec)	Day 2 (cm/sec)	Day 3 (cm/sec)	Day 4 (cm/sec)	Day 5 (cm/sec)
**Sham**	1	1	27.1	33.68	26.91	27.51	31.86
		2	33.45	25.66	24.06	28.22	34.79
		3	39.45	32.08	29.3	34.13	25.83
		4	33.45	24.16	15.66	22.03	28.17
	2	1	31	29.73	26.93	32.36	34.79
		2	32.32	27.77	24.58	24.11	32.81
		3	31.24	22.03	30.94	30.74	24.27
		4	34.25	38.56	27.25	27.65	27.33
	3	1	28.86	31.43	22.06	25.88	24.53
		2	31.68	30.24	28.78	26.94	28.68
		3	29.94	27.95	26.45	34.17	25.10
		4	28.5	38.67	28.81	32.19	25.82
	4	1	26.8	36.41	25.72	23.69	27.69
		2	25.76	32.64	23.89	37.16	24.61
		3	28.72	33.87	19.54	33.87	32.11
		4	29.45	30.23	29.46	27.51	37.40
	5	1	26.03	31.94	22.42	25.23	32.70
		2	32.81	30.48	22.92	17.46	37.17
		3	29.82	36.07	34.1	24.72	26.39
		4	30.77	35.21	31.22	38.09	24.22
	6	1	34.29	29.35	25.44	27.68	27.44
		2	28.85	32.97	25.08	26.91	25.23
		3	29.93	31.63	24.59	23.39	26.67
		4	40.29	31.54	24.69	19.34	41.09
	7	1	31.85	25.02	29.14	25.7	22.35
		2	28.15	20.22	28.28	27.06	32.01
		3	30.23	28.22	28.11	29.67	24.00
		4	33.65	25.33	23.29	27.2	34.24
	8	1	31.85	24.33	28.15	26.15	23.57
		2	30.82	31.4	14.87	23.35	25.16
		3	34.11	31.64	31.7	18.76	28.74
		4	30.95	29.66	26.56	26.6	34.46
	9	1	39.47	21.89	29.35	24.99	31.95
		2	31.9	38.05	31.41	27.89	30.93
		3	34.43	20.35	31.98	26.89	24.55
		4	29.65	25.97	32.84	26.97	30.09
	10	1	34.96	30.37	22.75	21.85	20.45
		2	32.29	27.38	31.29	29.09	26.16
		3	32.5	28.7	28.73	27.02	26.46
		4	29.6	23.43	32.87	26.62	29.56
			Mean ± SEM			28.79 ± 0.32	
**STZ**	1	1	39.65	30.82	29.65	32.56	21.6
		2	39.67	30.1	26.56	35.75	21.41
		3	34.18	27.47	28.37	21.7	18.74
		4	31.17	22.07	21.96	22.74	20.27
	2	1	25.26	37.26	32.5	29	18.74
		2	30.93	33.13	30.16	28.32	18.7
		3	33.21	27.16	24.4	37.66	17.53
		4	40.23	30.3	26	20.95	21.95
	3	1	36.56	22.32	32.52	25.89	32.15
		2	34.71	27.85	28.57	29.99	28.68
		3	35.89	27.42	30.08	29.65	24.11
		4	36.46	34.92	29.15	24.09	23.67
	4	1	33.91	23.07	30.18	23.27	31.98

EXCLI Journal 2018;17:999-1017 – ISSN 1611-2156
Received: August 16, 2018, accepted: October 05, 2018, published: October 29, 2018

		2	26.69	36.12	31.56	36.56	23.51
		3	41.12	30.79	31.39	34.12	23.46
		4	30.65	24.09	28.15	21.73	29.51
	5	1	32.43	26.02	30.82	29.56	30.86
		2	38.03	26.26	31.1	29.14	38.85
		3	37.33	21.32	36.84	23.43	29.18
		4	29.62	26.32	24.1	37.4	27.45
	6	1	34.46	35.45	28.83	23.59	28.65
		2	38.09	27.56	24.89	20.96	29
		3	30.55	39.83	27.53	33.66	36.26
		4	34.08	20.47	29.01	22.02	30.22
	7	1	28.58	29.27	31.61	28.66	27.68
		2	33.06	30.73	25.91	20.87	34.28
		3	33.45	22.55	27.38	35.68	38.78
		4	34.89	24.17	29.65	29.21	32.06
	8	1	34.63	24.29	31.63	31.23	28.06
		2	35.58	22.84	28.11	35.13	25.49
		3	32.74	27.4	31.52	27.63	24.13
		4	30.15	29.08	30.72	30.07	23.33
	9	1	33.71	23.65	21.06	28.11	30.92
		2	37.42	24.32	34.74	31.53	30.39
		3	41.23	30.05	30.32	31.03	31.52
		4	35.23	25.05	31.25	13.04	25.94
	10	1	27.25	23.01	26.2	27.71	28.84
		2	32.79	28.21	30.58	22.47	33.16
		3	32.01	22.45	26.09	21.2	27.98
		4	27.52	27.6	32.3	40.23	24.18
	11	1	35	27.46	27.69	37.26	26.01
		2	24.97	22.83	30.19	22.92	29.26
		3	30.72	27.52	30.12	22.73	28.1
		4	33.1	24.29	28.25	18.14	30.1
	12	1	30.23	26.63	23.95	21.09	25.92
		2	27.86	25.46	21.96	23.61	30.66
		3	28.18	23.38	28.11	31.42	35.37
		4	31.23	29.25	38.23	29.41	35.15
			Mean ± SEM			29.02 ± 0.33	
**STZ + EVR (1 mg/kg, 7 days)**	1	1	36.29	31.11	25.61	28.63	29.56
		2	33.64	35.32	30.61	20.82	28.21
		3	30.73	33.52	31.2	34.29	20.28
		4	25.36	28.71	24.94	24.45	27.88
	2	1	26.94	32.29	23.39	24.82	30.96
		2	33.55	32.4	20.74	26.74	19.33
		3	29.73	30.82	39.15	27.61	27.90
		4	22.29	27.8	34.62	34.20	19.86
	3	1	23.36	31.79	25.76	34.80	28.49
		2	11.29	30.98	30.03	31.96	28.54
		3	13.93	30.39	20.01	23.33	29.66
		4	30.36	26.64	37.39	22.71	37.72
	4	1	21.48	33.47	33.12	26.34	29.95
		2	10.4	34.56	37.24	33.66	23.75
		3	20.36	30.87	32.23	29.64	35.98
		4	30.31	27.38	29.25	29.25	22.19
	5	1	15.92	31.29	30.77	32.89	29.86
		2	25.97	17.78	28.94	30.52	31.79
		3	22.46	37.98	24.83	26.26	30.44
		4	27.62	22.89	16.37	27.47	28.92
	6	1	24.77	26.66	26.68	16.10	32.48
		2	30.74	27.7	29.85	23.46	26.33
		3	31.04	28.93	24.23	30.86	29.52

EXCLI Journal 2018;17:999-1017 – ISSN 1611-2156
Received: August 16, 2018, accepted: October 05, 2018, published: October 29, 2018

		4	31.39	25.93	20.93	25.48	24.25
	7	1	34.78	23.29	23.93	30.56	38.54
		2	32.9	23.08	30.69	37.18	17.74
		3	33.7	25.98	19.91	24.70	29.21
		4	32.42	28.12	26.48	18.37	37.99
	8	1	35.4	28.46	23.63	23.72	39.32
		2	38.1	33.96	24.37	22.06	20.74
		3	30.83	32.3	16.58	24.91	24.90
		4	30.71	29.71	17.94	38.06	35.07
	9	1	22.9	35.55	14.91	17.50	27.09
		2	39.58	27.39	18.5	35.54	21.37
		3	37.31	28.45	22.53	29.62	32.56
		4	35.48	25.52	28.72	36.09	29.71
	10	1	30.9	24	23.41	30.12	28.79
		2	39.74	16.16	28.67	23.08	13.54
		3	35.54	30.68	29	31.99	21.26
		4	33.25	24.31	25.44	14.81	34.51
			Mean ± SEM			27.96 ± 0.42	
**STZ + EVR (5 mg/kg, 7 days)**	1	1	31.02	31.54	30.29	26.89	20.73
		2	29.74	29.43	26.16	27.29	26.07
		3	21.65	30.11	37.49	22.34	33.58
		4	16.64	25.09	35.64	18.44	32.88
	2	1	26.69	31.63	26.12	28.22	33.58
		2	26.2	24.08	33.87	38.29	27.91
		3	39.72	39.68	23.68	30.43	24.54
		4	33.31	34.51	36.52	24.23	29.82
	3	1	26.07	28.54	35.08	23.31	34.64
		2	24.09	31.26	27.25	26.34	22.61
		3	28.71	31.29	25.92	28.05	35.85
		4	28.86	31.61	21.57	33.13	34.02
	4	1	20.82	24.08	25.52	21.71	29.78
		2	32.43	27.89	30.82	25.43	32.09
		3	24.09	26.47	37.61	34.3	30.34
		4	26.76	23.98	27.71	31.14	30.06
	5	1	21.89	24.04	27.73	20.98	34.32
		2	27.83	35.73	25.23	22.44	27.87
		3	33.35	31.25	35.93	32.28	33.81
		4	40.18	34.07	31.56	38.73	31.29
	6	1	26.1	28.12	24.72	23.19	29.89
		2	31.17	35.05	27.67	27.48	32.84
		3	27.11	32.81	33.72	28.98	24.58
		4	26.48	31.56	33.99	27.63	33.074
	7	1	30.49	21.38	22.19	29.56	21.63
		2	28.59	28.92	32.67	26.55	31.68
		3	28.23	32.61	37.69	23.7	20.78
		4	28.69	36.49	28.07	26.34	34.15
	8	1	33.97	23.17	28.95	25.51	25.28
		2	37.03	28.6	32.03	30.97	29.32
		3	35.23	29.93	35.22	29.63	37.05
		4	31.67	28.7	37.79	30.01	26.26
	9	1	33.51	29.87	34.06	23.66	8.44
		2	32.18	34.67	32.07	31.16	32.19
		3	32.84	30.13	31.75	30.01	17.93
		4	30.72	27.3	31.57	40.09	26.55
			Mean ± SEM			29.34 ± 0.37	
**STZ + EVR (1 mg/kg, 21 days)**	1	1	31.25	24.39	30.16	18.97	22.37
		2	29.45	29.63	33.62	17.46	31.80
		3	31.95	37.18	24.79	36.03	24.52
		4	30.81	23.49	33.85	30.55	36.46

EXCLI Journal 2018;17:999-1017 – ISSN 1611-2156
Received: August 16, 2018, accepted: October 05, 2018, published: October 29, 2018

	2	1	31.93	24.49	28.29	35.14	18.39
		2	37.98	28.58	36.70	28.12	24.75
		3	23.42	28.14	36.57	20.24	30.26
		4	33.56	23.31	30.27	28.26	26.96
	3	1	30.94	27.26	33.21	31.70	36.29
		2	38.01	26.70	31.87	29.65	33.85
		3	43.48	21.37	34.77	29.32	33.77
		4	39.56	20.11	32.04	24.36	37.33
	4	1	30.77	33.99	25.89	26.56	17.35
		2	30.72	31.85	32.29	25.36	38.60
		3	33.41	28.88	29.63	27.27	31.82
		4	38.42	39.78	26.70	19.95	38.67
	5	1	32.22	25.30	32.37	35.13	32.72
		2	41.49	39.18	22.82	26.62	28.27
		3	30.47	28.56	28.76	29.59	28.52
		4	35.03	32.16	23.41	28.80	34.79
	6	1	36.30	27.50	24.37	26.77	24.98
		2	29.94	40.73	28.45	25.05	26.17
		3	25.52	33.64	28.43	33.55	34.15
		4	23.49	36.30	21.90	37.60	32.10
	7	1	23.50	22.10	25.41	24.58	35.63
		2	23.76	30.79	29.64	38.84	24.58
		3	20.44	38.29	33.51	30.68	34.05
		4	22.06	37.78	26.04	28.20	31.32
	8	1	31.04	33.31	14.40	22.05	24.64
		2	28.82	30.17	33.32	29.69	27.15
		3	30.32	29.00	25.63	36.09	39.42
		4	29.16	21.81	29.91	27.83	25.71
	9	1	29.63	30.09	14.74	34.94	34.38
		2	25.76	27.62	18.97	27.13	26.20
		3	29.63	26.11	32.76	28.59	24.70
		4	36.00	25.71	33.46	25.14	38.79
	10	1	16.30	35.25	27.61	22.84	37.83
		2	19.83	33.62	27.06	30.90	29.10
		3	27.41	37.29	27.94	38.81	33.98
		4	31.61	31.57	24.76	26.42	29.31
			Mean ± SEM			29.61 ± 0.39	
**STZ + EVR (5 mg/kg, 21 days)**	1	1	27.84	24.76	38.14	32.36	27.64
		2	24.85	32.20	34.62	31.46	31.51
		3	21.95	34.60	30.69	31.90	35.97
		4	22.25	34.64	27.10	24.22	34.44
	2	1	28.41	29.28	34.40	33.03	26.11
		2	18.88	32.84	38.05	33.84	33.85
		3	26.02	29.08	28.23	24.04	36.66
		4	22.42	30.86	33.22	29.55	26.29
	3	1	29.47	25.71	21.71	22.33	24.60
		2	39.81	20.81	20.91	25.03	26.95
		3	35.11	28.39	20.90	28.51	22.97
		4	26.43	22.61	36.15	26.45	23.70
	4	1	25.16	26.36	37.13	27.59	25.90
		2	30.39	36.17	27.77	29.43	30.76
		3	38.64	35.16	29.23	38.19	35.95
		4	39.48	37.75	32.16	25.61	26.66
	5	1	30.54	27.62	22.27	32.88	26.98
		2	33.72	31.76	38.43	24.82	38.92
		3	32.86	35.55	31.69	36.09	29.53
		4	33.45	39.19	26.82	26.40	39.29
	6	1	30.93	23.47	31.11	18.64	21.88
		2	25.45	29.72	23.86	31.33	25.85

EXCLI Journal 2018;17:999-1017 – ISSN 1611-2156
Received: August 16, 2018, accepted: October 05, 2018, published: October 29, 2018

		3	25.79	25.20	24.77	37.20	33.90
		4	25.79	36.97	27.58	33.12	24.93
	7	1	24.03	28.34	24.13	31.00	25.10
		2	25.97	20.21	33.84	24.71	32.88
		3	26.42	24.23	25.91	31.03	28.19
		4	24.75	35.66	18.51	29.57	27.30
	8	1	24.79	31.27	27.21	23.05	28.73
		2	29.11	32.96	33.72	28.92	29.53
		3	25.93	24.05	32.03	35.59	30.05
		4	27.58	31.43	23.49	35.77	34.70
	9	1	23.00	33.04	35.40	29.45	33.06
		2	32.51	24.28	30.36	27.33	28.10
		3	33.34	27.18	18.61	31.38	27.36
		4	31.82	26.07	28.09	34.41	16.54
	10	1	24.90	32.29	33.89	30.45	21.33
		2	24.22	29.51	34.36	18.94	30.18
		3	26.88	29.34	30.56	34.18	29.07
		4	30.93	27.63	37.15	30.95	31.15
			Mean ± SEM			29.25 ± 0.35	
**EVR (5 mg/kg, 21 days)**	1	1	29.32	32.68	37.67	37.19	37.58
		2	29.98	33.09	28.82	26.49	30.58
		3	25.95	32.29	43.32	22.29	21.81
		4	21.77	38.52	30.93	26.29	23.08
	2	1	27.31	31.89	42.25	36.52	37.95
		2	35.95	31.70	22.39	22.91	29.16
		3	25.01	29.05	25.54	30.88	25.20
		4	25.88	23.40	21.44	30.25	39.47
	3	1	30.75	23.62	25.29	20.39	18.31
		2	35.82	19.77	17.83	25.09	30.75
		3	38.63	18.81	26.89	34.72	33.34
		4	30.65	19.09	26.08	31.58	32.74
	4	1	28.32	24.89	14.22	30.46	38.04
		2	36.50	22.84	35.58	28.39	30.36
		3	26.05	31.50	32.26	23.28	31.64
		4	26.38	46.80	21.72	32.33	32.55
	5	1	36.05	26.63	37.56	33.37	36.51
		2	32.98	27.91	48.67	17.93	31.58
		3	28.56	26.96	25.35	29.68	23.80
		4	35.28	35.93	33.14	37.90	21.01
	6	1	33.26	26.80	27.47	29.46	37.41
		2	35.20	26.71	38.61	29.59	32.11
		3	31.77	35.06	25.93	27.23	20.70
		4	29.39	27.09	30.39	22.49	29.44
	7	1	28.86	29.25	32.18	35.66	22.37
		2	23.20	31.14	34.84	31.70	25.09
		3	18.29	22.04	30.20	39.86	28.10
		4	17.02	18.41	25.45	28.12	26.41
	8	1	44.05	30.29	25.12	36.06	30.39
		2	25.88	33.18	29.71	21.83	27.03
		3	43.09	27.23	24.67	25.60	17.03
		4	35.06	26.22	28.01	33.41	27.91
	9	1	35.36	18.38	31.73	31.34	29.55
		2	28.73	32.22	22.31	32.58	21.09
		3	21.63	29.52	22.22	30.84	24.54
		4	27.23	38.76	30.64	33.41	31.14
	10	1	31.04	32.87	26.82	32.54	26.67
		2	31.30	28.04	26.45	14.69	33.46
		3	26.95	23.29	31.75	24.61	32.35
		4	17.37	40.06	24.70	27.42	29.42

EXCLI Journal 2018;17:999-1017 – ISSN 1611-2156
Received: August 16, 2018, accepted: October 05, 2018, published: October 29, 2018

Mean ± SEM	29.18 ± 0.43

EXCLI Journal 2018;17:999-1017 – ISSN 1611-2156
Received: August 16, 2018, accepted: October 05, 2018, published: October 29, 2018

Supplementary Table 5 (to Figure 6): Effects of 7 days or 21 days administration of EVR on malondialdehyde (MDA) level in the hippocampus of STZ-induced AD rats.

MDA Conc. (nmol/g tissue)

**Sham** N = 10	**STZ** N = 12	**STZ + EVR (1 mg/kg, 7 days)** N = 10	**STZ + EVR (5 mg/kg, 7 days)** N = 9	**STZ + EVR (1 mg/kg, 21 days)** N = 10	**STZ + EVR (5 mg/kg, 21 days)** N = 10	**STZ + EVR (5 mg/kg, 21 days)** N = 10
133.3	167.7	120.1	250.5	211.5	227	153.1
175.2	192	244.7	106.8	287.4	96.2	128.4
161.7	375.7	181.8	281.7	90.6	152	178.7
137.1	221.8	288.4	153.0	227.2	249.8	123.4
162.1	354.5	265.3	253.1	244.7	98.5	145.9
37.8	382.7	347.5	112.6	167.3	118.4	231
75.7	288.8	277.7	238.2	119.5	164.8	137.2
162.1	191.5	212.5	190.9	220.1	130	42.8
181.7	214.9	289.4	167.2	87.3	220.8	140.6
122.1	283.1	217.1		187.9	185	170.4
	323.8					
	289.7					
**Mean ± SEM**						
**134.9 ± 14.57**	**273.9± 21.8**	**244.5 ± 20.35**	**194.9 ± 21.4**	**184.4 ± 21.3**	**164.3 ± 17.41**	**145.2 ± 15.1**

EXCLI Journal 2018;17:999-1017 – ISSN 1611-2156
Received: August 16, 2018, accepted: October 05, 2018, published: October 29, 2018

Supplementary Table 6 (to Figure 7): Effects of 7 days or 21 days administration of EVR on total thiol level in the hippocampus of STZ-induced AD rats.

Total Thiol Conc. (mM)

**Sham** N = 10	**STZ** N = 12	**STZ + EVR (1 mg/kg, 7 days)** N = 10	**STZ + EVR (5 mg/kg, 7 days)** N = 9	**STZ + EVR (1 mg/kg, 21 days)** N = 10	**STZ + EVR (5 mg/kg, 21 days)** N = 10	**STZ + EVR (5 mg/kg, 21 days)** N = 10
0.42	0.27	0.16	0.19	0.38	0.24	0.32
0.54	0.12	0.16	0.25	0.48	0.40	0.42
0.43	0.03	0.21	0.31	0.18	0.46	0.34
0.52	0.08	0.37	0.42	0.29	0.29	0.55
0.34	0.17	0.17	0.46	0.38	0.22	0.40
0.20	0.26	0.06	0.29	0.28	0.54	0.40
0.29	0.30	0.09	0.45	0.35	0.48	0.15
0.53	0.28	0.33	0.16	0.35	0.33	0.57
0.24	0.10	0.19	0.31	0.24	0.40	0.42
0.44	0.07	0.19		0.46	0.48	0.36
	0.28					
	0.09					
**Mean ± SEM**						
**0.39 ± 0.04**	**0.17 ± 0.03**	**0.19 ± 0.03**	**0.32 ± 0.03**	**0.34 ± 0.03**	**0.38 ± 0.03**	**0.39 ± 0.03**

EXCLI Journal 2018;17:999-1017 – ISSN 1611-2156
Received: August 16, 2018, accepted: October 05, 2018, published: October 29, 2018

Supplementary Table 7 (to Figure 8): Effects of 7 days or 21 days administration of EVR on acetyl-cholinesterase (AChE) activity in the hippocampus of STZ-induced AD rats.

AChE activity (mmol/min/g protein)

**Sham**<br>N = 10	**STZ**<br>N = 12	**STZ + EVR (1 mg/kg, 7 days)**<br>N = 10	**STZ + EVR (5 mg/kg, 7 days)**<br>N = 9	**STZ + EVR (1 mg/kg, 21 days)**<br>N = 10	**STZ + EVR (5 mg/kg, 21 days)**<br>N = 10	**STZ + EVR (5 mg/kg, 21 days)**<br>N = 10
0.35	0.82	0.82	0.56	0.95	0.51	0.47
0.50	0.96	0.85	0.62	0.77	0.65	0.66
0.40	1.05	0.70	0.70	0.83	0.42	0.56
0.32	0.92	1.22	0.85	0.52	0.57	0.38
0.48	0.93	0.73	0.74	0.66	0.46	0.37
0.37	0.94	0.81	0.76	0.76	0.49	0.48
0.63	0.88	0.91	0.71	0.83	0.73	0.42
0.39	0.95	1.04	0.80	0.68	0.78	0.52
0.46	0.85	0.79	0.78	0.85	0.63	0.52
0.54	0.91	0.77		0.88	0.72	0.37
	1.19					
	0.82					
**Mean ± SEM**						
**0.44 ± 0.03**	**0.93 ± 0.03**	**0.86 ± 0.04**	**0.72 ± 0.03**	**0.77 ± 0.03**	**0.59 ± 0.03**	**0.47 ± 0.03**

Received: August 16, 2018, accepted: October 05, 2018, published: October 29, 2018

Supplementary Table 8 (to Figure 10): Effects of 7 days or 21 days administration of EVR on percentage of degenerated cells in the hippocampal CA1 region of STZ-induced AD rats.

Loss of cells (%)

**Sham**<br>N = 6	**STZ**<br>N = 6	**STZ + EVR (1 mg/kg, 7 days)**<br>N = 6	**STZ + EVR (5 mg/kg, 7 days)**<br>N = 6	**STZ + EVR (1 mg/kg, 21 days)**<br>N = 6	**STZ + EVR (5 mg/kg, 21 days)**<br>N = 6
9.1	93.8	69.7	22.2	47.6	42.8
13.6	56	25.7	9.5	17.2	8.1
10.8	94.2	42.2	41.9	26.4	3.1
7.3	83.6	59.5	23.6	42.4	8.8
4.7	93.8	52.6	24.3	9.1	13.4
6.6	33.3	72.3	67.9	20.9	17.9
**Mean ± SEM**					
**8.7 ± 1.3**	**75.8 ± 10.4**	**53.7 ± 7.2**	**31.6 ± 8.4**	**27.3 ± 6.1**	**15.7 ± 5.8**
